# Beta-Glucan as a Soluble Dietary Fiber Source: Origins, Biosynthesis, Extraction, Purification, Structural Characteristics, Bioavailability, Biofunctional Attributes, Industrial Utilization, and Global Trade

**DOI:** 10.3390/nu16060900

**Published:** 2024-03-21

**Authors:** Apurva Singla, Om Prakash Gupta, Vijeta Sagwal, Abhishek Kumar, Neha Patwa, Narender Mohan, Ankush, Dinesh Kumar, Om Vir, Jogendra Singh, Lokendra Kumar, Chuni Lal, Gyanendra Singh

**Affiliations:** 1 Barley Section, ICAR—Indian Institute of Wheat and Barley Research, Karnal 132001, Indiadinesh.kumar3@icar.gov.in (D.K.); omvir.singh2@icar.gov.in (O.V.); jogendra.singh2@icar.gov.in (J.S.); chuni.lal@icar.gov.in (C.L.);; 2 Division of Quality and Basic Sciences, ICAR—Indian Institute of Wheat and Barley Research, Karnal 132001, India

**Keywords:** β-glucan, cereals, fungus, microbes, bioavailability, biofunctionalities, industrial applications

## Abstract

This paper explores the multifaceted nature of β-glucan, a notable dietary fiber (DF) with extensive applications. Beginning with an in-depth examination of its intricate polysaccharide structure, the discussion extends to diverse sources like oats, barley, mushrooms, and yeast, emphasizing their unique compositions. The absorption and metabolism of β-glucan in the human body are scrutinized, emphasizing its potential health benefits. Extraction and purification processes for high-quality β-glucan in food, pharmaceuticals, and cosmetics are outlined. The paper underscores β-glucan’s biofunctional roles in immune modulation, cholesterol regulation, and gastrointestinal health, supported by clinical studies. The review discusses global trade dynamics by tracing its evolution from a niche ingredient to a global commodity. In summary, it offers a comprehensive scientific perspective on β-glucan, serving as a valuable resource for researchers, professionals, and industries exploring its potential in the dietary fiber landscape.

## 1. Introduction

Dietary fiber (DF) has long been recognized as a crucial component of a healthy diet, with numerous benefits for human health. Consumption of DF plays an important role in preventing and treating various health-related problems. Human gut microbiotas use these DFs as a substrate as these are not digested and absorbed by the human digestive system. DFs can be classified as soluble and insoluble on the basis of their physiological properties like solubilities, viscosities, and fermentability. Among the diverse array of DFs, beta (β)-glucan has emerged as a prominent and versatile soluble fiber with remarkable structural characteristics and a wide range of biofunctional attributes. Owing to increasing health consciousness and general awareness, the incorporation of DFs, including β-glucans in food, has now been gaining prime attention to get numerous preventive health benefits, including low-calorie, low-cholesterol, and low-fat. β-glucans are polysaccharides composed of glucose units linked by β-glycosidic bonds. Its unique structural properties, including branching patterns, degree of polymerization, and molecular weight, vary depending on its source. These structural variations play a pivotal role in determining the physicochemical properties and bioavailability of β-glucan, ultimately influencing its diverse biological effects. Owing to its ability to form viscous solutions, one of its major biofunctions is to act as a prebiotic, which significantly modulates digestion and absorption of different biomolecules, including micronutrients in the small intestine and presents various health benefits and food functionalities. Its origins span cereals, fungi, bacteria, and seaweeds, and its significance in human nutrition and industrial applications has sparked extensive research interest. In cereal grains, β-glucans are generally concentrated in the bran, the aleurone, and sub-aleurone layers [1]. Amongst cereals, barley and oat grains are considered to be the rich source of β-glucans compared to others, including wheat, rice, buckwheat, millet, and amaranth. 

The bioavailability of β-glucan, a key determinant of its physiological effects, depends on factors such as molecular weight, solubility, and the presence of other dietary components. Understanding the factors that influence β-glucan’s bioavailability is crucial for optimizing its health-promoting properties [2]. Not much progress has been made in the area of bioavailability assays of β-glucan either in vitro or in vivo. Therefore, looking into its significance in the food industry, extensive work on its in vitro and in vivo bioavailability is essentially required. Moreover, the extraction and purification processes of β-glucan from its natural sources have evolved significantly over the years, driven by advancements in technology and the need for higher purity and yield [3]. Here, the methodological advancement in β-glucan extraction and purification has been discussed in greater detail with a perspective on the method of choice both at the laboratory and industrial scale. Beyond its use as a DF, β-glucan exhibits diverse biofunctional attributes, including immunomodulatory, cholesterol-lowering, and prebiotic effects. These attributes have sparked intense interest in exploring β-glucan’s potential in various clinical investigations, shedding light on its efficacy in managing conditions such as diabetes, cardiovascular diseases, and gastrointestinal disorders. The basic biochemical and molecular mechanism of its action on different health conditions has been discussed in greater detail. In the realm of industrial utilization, β-glucan has found applications in a wide array of products, ranging from functional foods and dietary supplements to pharmaceuticals and cosmetics. Its functional properties, such as thickening and gelling abilities, contribute to its versatile role in product formulation and development [4]. Furthermore, the global trade of β-glucan has witnessed significant growth, driven by increasing consumer awareness of its health benefits and its incorporation into a variety of consumer products. Understanding the dynamics of this global trade is essential for industry stakeholders, policymakers, and researchers alike.

As societies worldwide grapple with the increasing burden of diet-related health issues, the exploration of β-glucan as a soluble DF source has gained momentum. This comprehensive review aims to provide an in-depth exploration of β-glucan, encompassing its structural characteristics, diverse origins, bioavailability in the human body, extraction, and purification techniques, multifaceted biofunctional attributes, clinical investigations highlighting its health benefits, industrial applications, and its place in the global trade market. By synthesizing the latest scientific findings and industry trends, this review intends to offer a holistic perspective on the multifaceted role of β-glucan in nutrition, health, and commerce, shedding light on the significant strides made in understanding and harnessing the potential of this remarkable soluble DF. 

## 2. Potential Sources of β-Glucan 

The major sources for β-glucan as a valuable functional ingredient include cereals like oats, barley, sorghum, wheat, rye etc. Among these, oats and barley share a satisfactory and chemically similar polysaccharide β-glucan, i.e., (1→3), (1→4)- mixed linkage β-D-glucan. β-glucan content may vary from cultivar to cultivar under specific environmental conditions. Therefore, depending on the specific variety, oats contain 6–8% and barley 4–10% (*w*/*w*) β-glucan [5]. Different sources of β-glucan and their molecular characterization, along with their DP3: DP4 ratio, are given in Table 1. The day-to-day consumption of 3 g of β-glucan has been shown to significantly reduce the level of cholesterol concentrations in blood. It also reduces the circulation of low-density lipoproteins (LDL), which are considered to be the key risk factors for cardiovascular diseases [6]. The efficient daily dose of 3 g β-glucan can be obtained from 75 g of whole grain oats (minimum 5.5% β-glucan) or 55 g of oat bran (4% β-glucan). Additionally, some mushroom varieties are reported to be a good source of β-glucan such as the oyster and shiitake mushrooms. Mushroom cell walls are rich in long or short-chain polymers of glucose subunits with β-1,3 and β-1,6 linkages that are responsible for the linear and branching structures of β-glucan [7]. It is worth mentioning that an extract of the edible oyster mushroom reduced the release of tumor necrosis factor-α (TNF-α) and interleukin-6 (IL-6) by lipopolysaccharide (LPS)-challenged monocyte in vitro, and also, in mice challenged with LPS (in vivo) after administration of the mushroom extract [8]. Mushroom β-glucans remain undigested in the human gastrointestinal tract, reaching the bowel virtually unchanged. It forms a gel at the mucosa surface and modulates biliary salt resorption, ultimately modifying the gut microbiota [7]. Additionally, fragments of β-glucans found in the serum are absorbed from the intestinal tract.

## 3. Advances in the Biosynthetic Pathway of β-Glucan 

**Figure 1:**
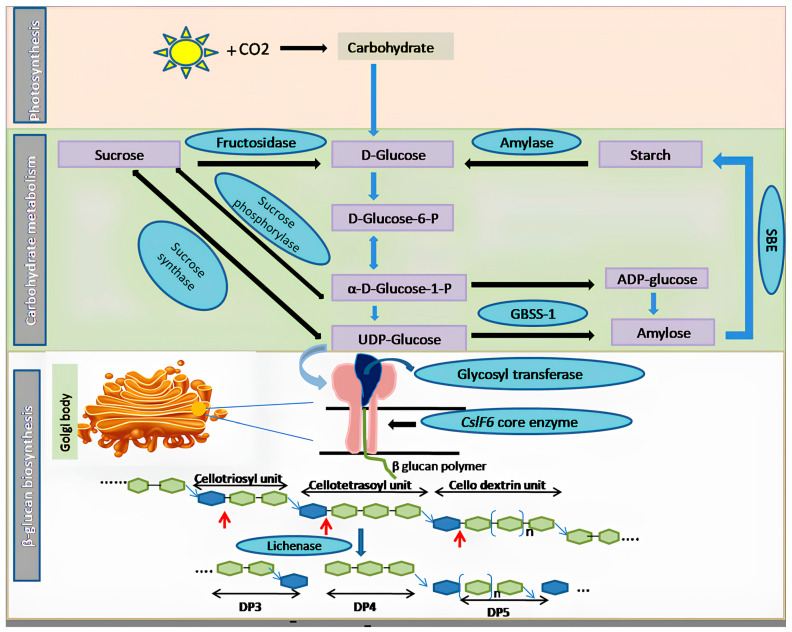
Schematic presentation of the β-glucan biosynthetic pathway in cereals. CO_2_: carbon dioxide, D-Glucose-6-P: D-Glucose-6-Phosphate, α-D-Glucose-1-P: α-D-Glucose-1-Phosphate, UDP-Glucose: uridine diphosphate, SBE: starch branching enzyme, GBSS-1: granule bound starch synthase-1, Cslf6: Cellulose synthase-like family 6, DP: degree of polymerization. Red arrows indicate the site of action of the lichenase enzyme.

Recent research highlights the dynamic nature of β-glucan levels, which tend to increase during the growth and developmental phases in plants but decrease once growth ceases. In specific cereals like barley, oats, and rye, β-glucan is primarily localized in the aleurone layer, where it plays a pivotal role in germination [19]. During germination, the enzyme β-glucanase assumes a critical role by catalyzing the breakdown of β-glucan molecules, thus providing an essential source of carbohydrates for germinating seeds. The intricate biosynthesis of β-glucan involves a multifaceted network of enzymes and genes closely related to both starch and cellulose pathways. Notably, members of the cellulose synthase-like (CSL) subfamilies CSL A to H and J have been implicated in β-glucan biosynthesis [20,21] (Table 2). A schematic representation of the β-glucan biosynthesis pathway is shown in Figure 1. Central to this pathway is the enzyme CSL, which catalyzes the formation of cellobiosyl units through the linkage of two glucose residues. Subsequently, a glycosyl transferase enzyme transfers glycosyl residues onto cellobiosyl, forming cellotriosyl units and other odd-numbered units. Extensive studies have underscored the crucial role of *cellulose synthase-like F*(*CslF*) genes in β-glucan biosynthesis. Gain-of-function experiments with transgenic approaches and loss-of-function experiments using RNA interference (RNAi) have demonstrated their significance. Among these genes, CslF6 stands out as the primary gene responsible for β-glucan synthesis. For instance, Ref. [22] reported the direct involvement of *HvCslF6*, *HvCslH1*, and *HvCslJ* in β-glucan biosynthesis by generating mutants with reduced *Cslf6* expression. Moreover, in barley, CRISPR/Cas9-induced mutations in *CslF6* and *CslH1* genes revealed their critical roles in cell wall polysaccharide synthesis. Frame shift mutations in these genes led to alterations in polysaccharide content and grain characteristics, further highlighting the importance of *HvCslF6* [23]. 

In a notable advancement, Ref. [28] introduced *CslF2* and *CslF4* genes from rice into Arabidopsis, which typically lacks β-glucan in its cell wall. This genetic manipulation resulted in the synthesis of β-glucan in Arabidopsis cell walls, substantiated by enzymatic assays and specific antibodies. [27] further confirmed the role of *CslF6* in mixed linkage β-glucan biosynthesis in rice. Intriguingly, loss-of-function mutations in β-glucan synthesis genes in rice were found to enhance disease susceptibility, underscoring the critical role of these genes in bolstering the immune and defense systems [28]. Work conducted by [30] has provided valuable insights into the biochemical characteristics and spatial organization of β-glucan in cereals and grasses. Their work elucidated distinct synthesis patterns for various cell wall polysaccharides, with cellulose synthesis occurring at the plasma membrane and mixed polysaccharides being synthesized within Golgi bodies. Importantly, *CslF6* was identified as the key enzyme responsible for generating cello-dextrins, which were subsequently linked at the plasma membrane, forming β-D-glucan chains. Further research by [31] identified seven genomic regions in tetraploid wheat associated with β-D-glucan content. Marker trait association (MTA) analyses across various grass species implicated putative candidate genes in the modulation of β-glucan synthesis, particularly in the context of carbon resource partitioning. Additionally, [20] expanded our understanding of β-glucan biosynthesis by identifying 14 MTA and seven genes encoding enzymes involved in glucose metabolism linked to β-glucan production in barley. Transcriptome studies revealed differentially expressed genes associated with hydrolase activity, starch synthesis, and β-glucan metabolism, all of which are intricately linked to the process of β-glucan biosynthesis [21]. In vitro experiments have unequivocally demonstrated that UDP-glucose, in the presence of Mg^2+^, serves as a precursor for β-glucan synthesis, catalyzed by the CslF6 enzyme. The lichenase enzyme was employed to hydrolyze the 3^H^-labeled polymer to confirm the linkage between glucosyl subunits within the synthesized polymer. This enzymatic digestion yielded DP3 and DP4 fragments, indicating specific cleavage of β-1,4 linkages followed by β-1,3 linkages, thereby providing concrete evidence for the role of the *CslF6* gene in β-glucan synthesis. 

**Figure 2:**
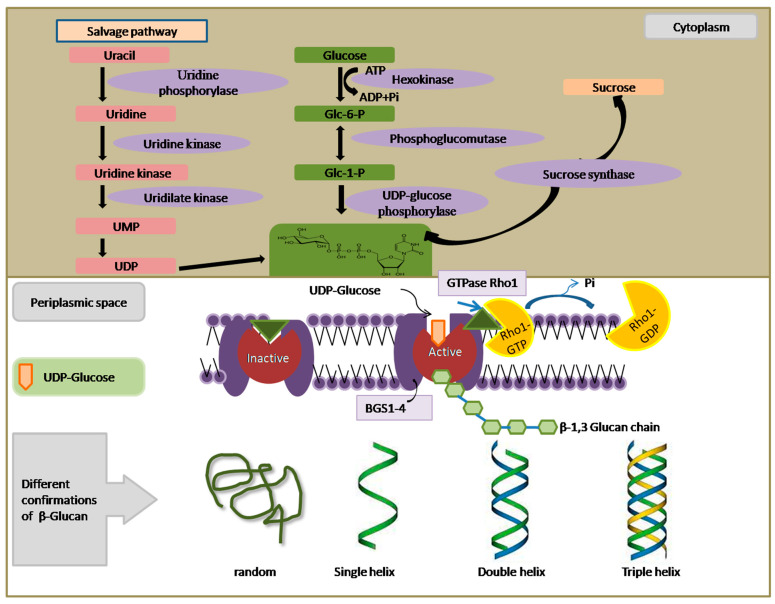
Model representing the β-glucan biosynthetic pathway in yeast. UMP: uridine monophosphate, UDP: uridine diphosphate, Glc-6-P: Glucose-6-Phosphate, Glc-1-P: Glucose-1-Phosphate, BGS: β-glucan synthase, ATP: adenosine triphosphate, ADP: adenosine diphosphate, GTP: guanosine triphosphate, GDP: guanosine diphosphate, Pi: inorganic phosphate, Rho 1: Ras like GTP binding protein.

Other than the plant kingdom, β-Glucan is primarily found in the cell wall and division septum of yeast. Its principal role lies in providing structural rigidity and fortifying the cell wall. Immunoelectron microscopy has revealed the accumulation of β-(1,6)-glucan particles associated with the Golgi apparatus in the yeast species *Schizosaccharomyces pombe*. This suggests that the binding of β-(1,3)-glucan occurs at the cell surface, whereas the initial production of β-(1,6)-glucan takes place in the Golgi. In contrast to cereals, where the enzyme CslF6 is responsible for β-(1,3)-glucan synthesis, yeast employs an enzyme complex known as β-(1,3)-glucan synthase (BGS), which is situated on the cytosolic face of the plasma membrane. BGS catalyzes the production of β-(1,3)-glucan using UDP-glucose as a substrate (Figure 2). In vitro experiments have demonstrated that this enzyme generates linear chains of approximately 80 glucose molecules, which are subsequently elongated and branched in yeast species like *Saccharomyces cerevisiae* with the aid of glycosylphosphatidylinositol (GPI)-bound Gas proteins. These proteins belong to the glycosidase/transglycosidase 72 family. Several other genes are involved in the biosynthesis of β-glucan in yeast, as outlined in Supplementary Table S1. An in vitro assay has been employed to identify the subunits of BGS, where acid-insoluble radioactive β-(1,3)-glucan accumulates when membrane extracts are treated with radiolabeled UDP-glucose and GTP. A similar experiment was performed to separate BGS into membrane-bound and cytosolic components, which respectively carry the catalytic and regulatory GTP-binding modules [32]. Fission yeast possesses four genes, namely *bgs1* to *bgs4*, encoding four putative BGS catalytic subunits. Among these, *bgs1*, *bgs3*, and *bgs4* are essential for vegetative growth, while *bgs2* is involved in spore wall formation and sexual differentiation. A critical regulatory component in this process is the GTPase Rho1, a Ras-like GTP-binding protein that activates the BGS complex by binding to GTP. Collectively, findings from various organisms strongly support the role of Bgs proteins in catalyzing β-(1,3)-D-glucan chain biosynthesis in vivo. However, it is worth noting that none of these subunits possess the predicted UDP-glucose binding consensus sequence (R/K)XGG. Furthermore, BGS has not yet been isolated and uniformly purified from the plasma membrane, so it remains uncertain if reintroduced, pure BGS is capable of forming in vitro β-(1,3)-D-glucan chains.

In essence, β-glucan biosynthesis represents a complex yet finely orchestrated process with significant implications for plant growth, development, and defense mechanisms. The multifaceted interplay of enzymes and genes, along with their roles in the synthesis and modulation of β-glucan, continues to be a focal point of research in the field of plant biology. These findings shed light on fundamental processes within plants and hold promise for potential applications in agriculture and nutrition. Future research in the field of β-glucan biosynthesis promises to explore several exciting avenues. Firstly, there is a growing need to unravel the intricate regulatory mechanisms governing β-glucan production in cereals, with a focus on genetic and epigenetic regulation. Secondly, advancements in genome editing technologies like CRISPR/Cas9 offer the potential for precision engineering of β-glucan content and structure in crops, with implications for enhanced nutritional value and industrial applications. Furthermore, understanding the impact of β-glucan manipulation on plant growth, development, and immunity will expand our knowledge of plant biology. In the case of yeast, ancillary evidence from purified active membrane-bound subunits will be essential for demonstrating without a shadow of a doubt that the Bgs/Fks proteins are the BGS catalytic subunits and to provide concrete proof of how each Bgs protein uniquely biosynthesizes a different β-D-glucan.

## 4. Methodological Advances in Extraction and Purification of β-Glucan from Different Sources

### 4.1. Advances in Extraction Procedure

**Figure 3:**
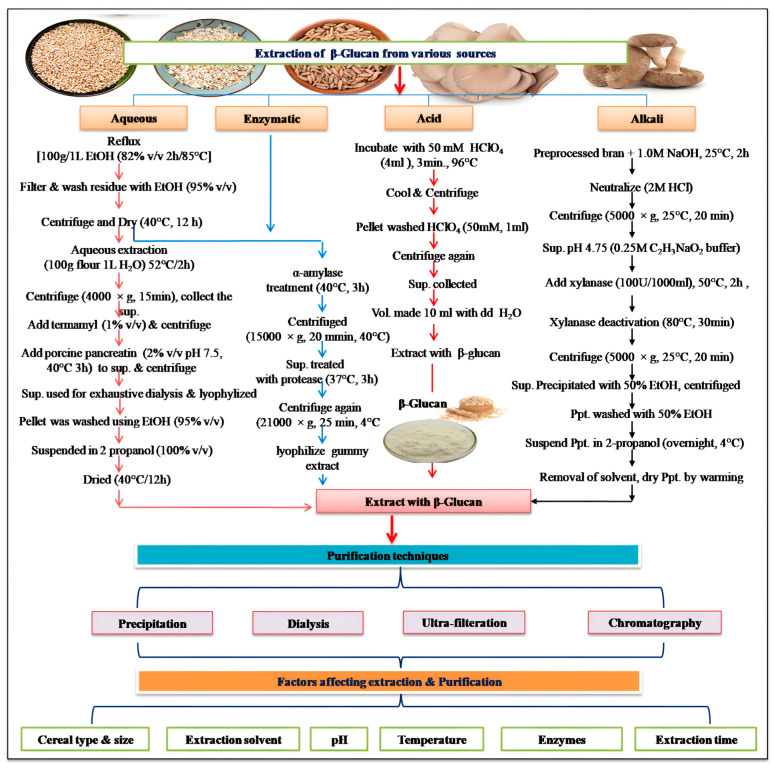
Extraction and purification of β-Glucan, along with the factors influencing their extraction and purification. Abbreviations: EtOH: ethanol, HClO_4_: perchloric acid, Vol.: volume, NaOH: sodium hydroxide, HCl: hydrochloric acid, C_2_H_3_NaO_2_: sodium acetate, dd H_2_O: double distilled water, Ppt.: precipitates, Sup.: supernatant.

The attainment of high-purity β-glucan is a paramount concern for both laboratory and commercial applications, with a keen focus on factors like purity, molecular integrity, and yield. The method of extraction significantly influences the molecular weight and structural characteristics of β-glucan. Variations in estimates of β-glucan molecular weight may arise from extraction and purification procedures, aggregation phenomena, and depolymerization events during the extraction process [33]. Therefore, the extraction methods should preserve β-glucan molecule integrity while maximizing both yield and purity to assess the structural attributes of isolated β-glucans. Since its discovery, numerous extraction techniques have been developed and optimized to maximize yield. Common wet techniques for β-glucan extraction include aqueous, alkaline, acidic, and enzymatic methods. Among these, hot water extraction is the most widely adopted. However, wet extraction methods suffer from drawbacks such as lengthy extraction durations, high operational costs, and environmental concerns. Wet methods utilize water, acidified water, or alkali to solubilize β-glucan by hydrolyzing whole grains like barley or oats. Following solubilization, centrifugation and ethanol precipitation are employed to separate the β-glucan from the slurry. Ethanol treatment may inhibit native enzymes, potentially removing nonpolar molecules and dissociating proteins and sugars. Subsequent to the initial rough extraction, further refinement is performed to eliminate contaminants like starch, protein, and fat, among others. Various extraction and purification techniques of β-glucan are laid out in Figure 3. On the other hand, dry separation techniques like pearling and air classification are commonly used to avoid the use of solvents. However, dry techniques typically yield less β-glucan recovery (<30%) compared to wet methods, which can achieve a higher β-glucan recovery ranging from 20 to 70% [34].

A commercially viable and environment-friendly extraction procedure of β-glucan would overcome the aforementioned problems. Recently, two new extraction techniques, namely superheated water extraction and rapid solvent extraction, have been developed that are commercially viable and environment-friendly with increased β-glucan yield. The selection of the appropriate extraction technique is contingent upon various factors, including the source of raw materials, desired purity levels, and cost considerations (Supplementary Table S2). Diverse cereals exhibit varying β-glucan content and extraction efficiency, with barley and oats reigning as the premier β-glucan sources, followed by wheat, rye, and corn [6]. Particle size is a critical determinant, as reduced particle dimensions provide heightened surface area exposure, thereby augmenting extraction efficiency. Selection of the extraction solvent hinges on the β-glucan type and purity goals, with water, ethanol, and acetone serving as prime candidates [35]. pH modulation of the extraction solution profoundly influences β-glucan solubility, with alkaline solutions surpassing acidic counterparts in efficacy. Temperature adjustment plays a pivotal role, impacting extraction rates while necessitating prudent regulation to prevent β-glucan degradation. Enzymatic content within the extraction solution demands inactivation through methods such as heat treatment or enzyme inhibitors. Extraction time and duration significantly influence β-glucan yield, with longer durations often yielding higher quantities, albeit at the risk of potential β-glucan damage. Notably, the solubilized β-glucan is susceptible to molecular fragmentation resulting from shear forces during mixing and centrifugation, as well as enzymatic degradation catalyzed by the inherent β-glucanases in the aqueous system. This enzymatic breakdown and shear-induced fragmentation lead to reductions in the molecular weight and viscosity of β-glucan. Unfortunately, this diminishes the potential cholesterol-lowering and hyperglycemia-attenuating effects of β-glucan. Below, we delve into a detailed discussion of some commonly employed techniques for β-glucan extraction.

#### 4.1.1. Aqueous Extraction

Aqueous extraction is a commonly used method for isolating β-glucan from cereals, characterized by its simplicity and cost-effectiveness. This process involves grinding cereals into a fine powder, which is then mixed with water. The mixture is heated to a temperature typically within the range of 50 to 60 °C. Subsequently, insoluble components are removed through filtration, and the β-glucan is concentrated via evaporation. The temperature of the aqueous extraction process plays a pivotal role in β-glucan recovery. Ref. [36] observed a positive correlation between temperature and β-glucan yield, with a maximum extraction efficiency of up to 89.1%. Consequently, the use of thermally stable α-amylase to hydrolyze starch during hot water extraction, as suggested by [36], becomes relevant. In the case of barley, β-glucan extraction employs water as the solvent. Alternatively, Ref. [37] explored a method for β-glucan extraction from oats and barley, involving cereal grain grinding to create flour, which is then mixed with water to produce a slurry containing an aqueous β-glucan solution and solid residue. The aqueous solution is separated from the solid residue, and water is removed through evaporation, ultrafiltration, or a combination of methods to obtain a gel or solid enriched with β-glucan. Despite the advantages of aqueous extraction, such as its speed, simplicity, and affordability, it is essential to acknowledge its limitations. This method is generally less efficient and may compromise the biological activity of β-glucan compared to alternative techniques. Therefore, further research is needed to optimize extraction procedures to achieve both maximum recovery and enhanced biological activity.

#### 4.1.2. Alkaline, Acidic and Enzymatic Extraction

Over the years, the extraction of β-glucan has witnessed several advancements, with alkaline extraction gaining prominence over aqueous extraction due to its increased effectiveness. However, it carries the potential drawback of compromising the structural integrity and biological activity of β-glucan. This method is often costlier than aqueous extraction and necessitates the use of inorganic chemicals such as potassium or sodium hydroxide. The procedure involves prolonged stirring, followed by filtration to eliminate insoluble components and evaporation for β-glucan concentration [38]. In contrast, acidic extraction is a less potent but gentler method that is less likely to harm β-glucan during extraction. It involves mixing finely ground cereal raw materials with an acidic solution containing sulfuric or hydrochloric acid. The mixture is heated within a temperature range of 50 to 60 °C, with continuous stirring, followed by filtration to remove insoluble fractions. This process is more cost-effective than enzymatic extraction and carries a lower risk of β-glucan damage [39]. Among the extraction techniques, enzymatic extraction is considered the most effective, yet it is also the costliest. This method entails mixing grain powder with an enzyme that selectively breaks down β-glucan without significantly altering its original structure. However, it is primarily suitable for small-scale experimental purposes. In recent years, alternative methods such as accelerated solvent extraction (ASE), reflux extraction, microwave-aided extraction (MAE), and ultrasound-assisted extraction (UAE) have emerged for extracting high-quality β-glucan from various cereal sources [37]. ASE, in particular, offers a more environmentally friendly and efficient approach, yielding higher β-glucan quantities in a shorter extraction time. Collectively, these advances have aimed to enhance the extraction and preservation of β-glucan from diverse cereal sources. Nevertheless, the quest for a robust, cost-effective, reproducible, and eco-friendly industrial-scale extraction method remains an intriguing challenge for the future.

### 4.2. Advances in Purification Procedure 

Recent advancements in the purification of β-glucan have propelled this field to new heights. These innovations have revolutionized the separation and refinement of β-glucan, contributing to its increased purity and suitability for a wide range of applications. Researchers have leveraged cutting-edge techniques and technologies to enhance the efficiency and precision of β-glucan purification, addressing challenges associated with impurities and yield. In this context, we will explore the key breakthroughs that have transformed the purification procedure of β-glucan, unlocking its full potential in various industries. The purification of β-glucan often necessitates the use of a suitable solvent to induce precipitation, with ethanol being the predominant choice due to its miscibility with water. This precipitation process unfolds in two sequential steps: the initial dissolution of β-glucan in a solvent, followed by the congregation and precipitation of β-glucan molecules due to the nonpolar nature of the solvent. Centrifugation or filtration is subsequently employed to isolate the precipitate from the solution [35]. The choice of solvent for precipitation hinges on the solubility of β-glucan; when water fails to dissolve it, ethanol or acetone becomes the alternative. Nonetheless, water remains the quintessential solvent for β-glucan precipitation. The effectiveness of the precipitation process is directly influenced by the concentration of β-glucan in the solution, as higher concentrations enhance the likelihood of successful precipitation. This method stands out for its expeditious and cost-effective nature, proficient in ridding β-glucan solutions of various impurities, although time constraints and the potential risk of β-glucan degradation may limit its application.

Dialysis emerges as an alternative purification technique, segregating molecules based on size. In the context of β-glucan purification, dialysis entails passing the solution across a semipermeable membrane that selectively permits the passage of small molecules and water while obstructing β-glucan. Cellulose acetate membranes are widely employed and renowned for their impermeability to β-glucan yet permeability to water and small molecules. Dialysis serves as a gentle method for the elimination of contaminants from the β-glucan solution, with ethanol and ammonium sulfate precipitation commonly applied as a follow-up step [40]. Moreover, dialysis has the capacity to extract glucose, peptides, and amino acids from β-glucan, elevating the final product’s purity. Although dialysis is time-consuming and may not be suitable for large-scale β-glucan production, it delivers a product characterized by superior purity and viscosity when compared to alternative methods. Nevertheless, its adoption necessitates additional time and specialized equipment. Notably, ultrafiltration employing semipermeable membranes with pore sizes ranging from 250 to 500 nm has emerged as a time-saving approach. The application of strong shear forces in this method has led to reduced viscosity and molecular weight of β-glucan. Among several techniques explored, including isoelectric precipitation, solvent extraction, deproteinization with active carbon, and alcohol precipitation, a combination of alcohol extraction with the ammonium sulfate approach stands out, delivering a remarkable 91.38% purity despite a moderate yield [33]. Chromatography has emerged as a prominent method for β-glucan purification, offering meticulous separation of its constituents. Gel filtration chromatography is a favored choice, with columns filled with porous gel matrices of varying sizes. β-glucan molecules selectively adhere to these matrices, contingent on their size, resulting in a sequential elution of smaller and larger molecules. This technique is highly effective in enhancing β-glucan purity. However, it is imperative to acknowledge that chromatography, while efficient, presents challenges in terms of time and cost. Furthermore, chromatography, as a more intricate and rewarding approach, involves suspending the β-glucan solution within a solid matrix column. The specific binding of solution components to the matrix facilitates subsequent separation through solvent elution. A range of chromatographic methods, such as gel filtration, ion exchange, and affinity chromatography, can be tailored to the unique characteristics of β-glucan and the desired purity levels. It is important to note that despite its high efficiency, chromatography demands significant resources, and the nature of impurities significantly influences the choice of the most suitable purification strategy.

Recent innovations in β-glucan purification have significantly improved purity and versatility across industries—cutting-edge methods like precipitation, dialysis, ultrafiltration, and chromatography address impurity and yield challenges. Precipitation offers cost-effective speed, dialysis provides superior purity, ultrafiltration reduces viscosity, and chromatography ensures precise separation. Nonetheless, these methods entail time, cost, and resource considerations. Future perspectives in β-glucan purification revolve around optimizing existing methods for scalability and cost-effectiveness and exploring innovative approaches, including nanotechnology and advanced chromatographic techniques, to further elevate purity and yield. The integration of these techniques with sustainable practices and green chemistry principles is also a promising avenue to enhance the environmental sustainability of β-glucan production and purification processes. Additionally, research into the development of tailored purification strategies based on the unique characteristics of specific β-glucan types and application requirements will be pivotal in unlocking the full potential of this versatile polysaccharide.

## 5. Deciphering Structure-Function Characteristics and Types of β-Glucan

**Figure 4:**
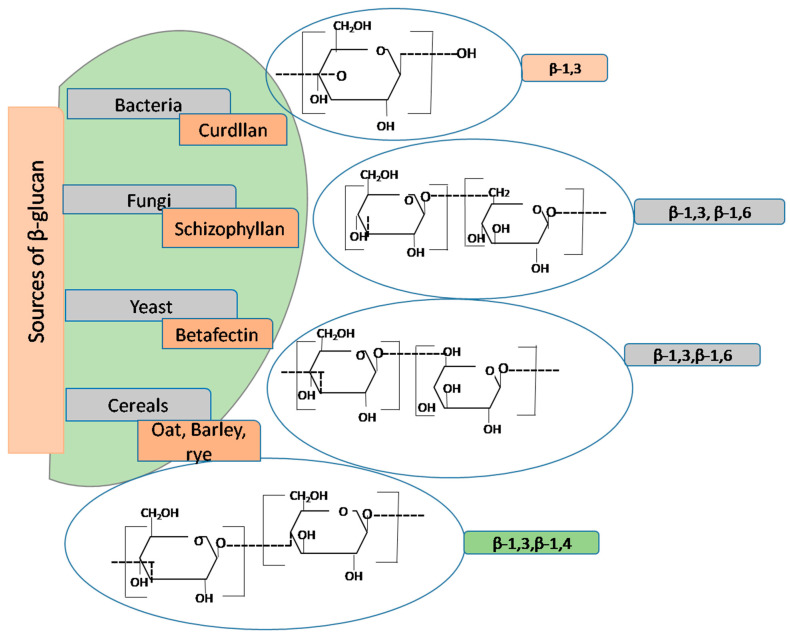
Schematic model representing structure and branching degree of β-glucan from different sources.

β-glucan has garnered recognition as a bioactive food ingredient due to its diverse biological activities. It is ubiquitously found in various sources, including bacteria, algae, barley, yeast, mushrooms, and oats. The structural intricacies of β-glucan molecules, as illustrated in Figure 4, reveal a spectrum of variations depending on their origin. These polymers consist of glucose subunits linked by β-(1,3) or β-(1,4) glycosidic bonds, often with branching occurring at the six-position of the backbone. For instance, mushroom-derived β-glucans typically feature short β-(1,6)-linked branches, while yeast-derived counterparts exhibit β-(1,6)-side branches along with additional β-(1,3) regions [41]. β-glucans possess a remarkable amalgamation of attributes, combining the functional properties of viscous and gel-forming food hydrocolloids with the physiological benefits of DFs. The exploration of β-glucan’s properties dates back to the 1950s, with pioneering studies focusing on dried yeast β-glucan extracted from *Saccharomyces cerevisiae*. Characterization of its structure relied on comprehensive analyses of total sugar content and molecular weight, employing advanced techniques such as multi-angle laser light scattering in conjunction with size exclusion chromatography [42]. 

Fungal species are characterized by the prominent presence of chitin and β-glucan in their cell walls and intracellular structures. The arrangement of cellodextrin, a vital component, is intricately linked to the concentration of substrate UDP-GluP. When UDP-GluP concentrations are low, glycosyl transferase activity is hindered, thereby impeding the transfer of glucose residues to the cellobiose unit [30]. The molecular weight of β-glucan, along with its water retention properties and solubility, profoundly influences viscosity. Solubility hinges on the molecular weight of β-glucan, which can vary widely depending on the source and extraction method. Studies have illuminated the molecular weight of oat and barley β-glucans, spanning from 130 kDa to 390 kDa and 190 kDa to 410 kDa, respectively. Notably, some β-glucans exhibit structural deviations, characterized by different branching patterns, linkages, or attachment of other molecules, such as proteins, as seen in polysaccharide-K (PSK, Krestin) and polysaccharo-peptide. β-glucan, composed of β-D-glucose monomer units connected by glycosidic linkages at positions β (1→3), (1→4), and/or (1→6), either in branched or unbranched configurations, holds significant promise in promoting health. Its beneficial effects include reducing the glycemic index, managing cholesterol levels, and mitigating various cardiovascular diseases. The molecular weight, shape, and structure of β-glucans, influenced by isolation techniques, play a pivotal role in triggering various biological activities, particularly immunological responses. In aqueous solutions, β-glucans adopt triplex helix, singlet, or duplex conformations, with the immune potency believed to hinge on conformational complexity and molecular weight. To harness the full spectrum of activities and qualities inherent to β-glucan, an increasing number of research endeavors have delved into innovative strategies to modify its conformation. These approaches include physical methods involving temperature variation, chemical methods employing acids, alkalis, and salts, enzymatic methods utilizing enzymes like glucanase and lichenase, and mechanical techniques such as homogenization and sonication. These avenues of exploration hold promise for optimizing the potential applications of β-glucan in diverse fields.

When subjected to enzymatic digestion with lichenase, β-glucans yield oligomeric products, including trisaccharide units (degree of polymerization; DP-3) known as cellobiosyl-D-glucose, tetrasaccharide units (DP-4) termed cellotriosyl, and extended cellulose-like cellodextrin units (DP > 5). These cellodextrin units have a propensity for aggregation, precipitating out of the β-glucan chain, thus posing challenges in quantitative analysis. These oligosaccharides may exist in a random or non-random arrangement within the β-glucan chain. It is noteworthy that cereals, despite sharing a similar molecular structure, exhibit variations in the ratio of DP3 to DP4 and the ratio of cellotriosyl to cellotetrosyl and their molecular size. For example, wheat demonstrates a higher ratio of DP3/DP4 (3–4.5), while barley presents a lower ratio (2.4–2.5), and oats fall between 1.6 and 2.3 [43]. Environmental factors, including growth conditions, can exert influence over the degree of polymerization and the DP3:DP4 ratio. Furthermore, the molecular weight of β-glucan can be influenced by environmental constituents, extraction, purification methods, and genetic factors. This variation in molecular weight is a critical determinant of β-glucan’s physical properties, such as viscosity and solubility, which in turn play a pivotal role in shaping its biofunctionalities. Understanding these intricate structural nuances is fundamental in appreciating the diverse health implications of β-glucan and optimizing its utilization in various applications.

Lentinan, a β-glucan derived from the fungus *Lentinus edodes*, demonstrates a remarkable structural characteristic: it forms triple helical structures at room temperature. This unique property imparts to it a high viscosity and an exceptional ability to withstand a wide range of environmental conditions, including pH, temperature, and salt concentrations, when dissolved in aqueous solutions. This feature alone makes lentinan a subject of keen interest for researchers and industry professionals alike. [44] shed light on the intriguing conformational diversity within β-glucans. They found that high molecular weight β-glucans, like schizophyllan, can adopt both single and triple helical conformations. In stark contrast, low molecular weight β-glucans tend to exhibit a random coiled conformation. These molecular and structural characteristics are of paramount importance, as they fundamentally influence the physical properties of β-glucans, such as their solubility and rheological behavior, and their functional effects when incorporated into food products. Low molecular weight yeast β-glucans exhibit superior antioxidant and immunological activities. Conversely, [45] found that higher molecular weight β-glucans sourced from *Chlorella pyrenoidosa* display potent immunostimulatory properties. These findings underscore the intricate relationship between β-glucan structure and its biological effects. Within the food industry, β-glucans are extensively utilized due to their unique gelling properties, which enhance the viscosity of aqueous solutions. This, in turn, contributes to improving the texture and appearance of a wide range of food products, including salad dressings, gravies, and ice creams. Moreover, β-glucans have found utility as fat mimetics, facilitating the development of calorie-reduced products. Intriguingly, the multifaceted properties of β-glucans, encompassing their structural diversity, functional effects, and versatile applications, continue to captivate the interest of researchers, food scientists, and industry professionals. This dynamic interplay between molecular structure and functionality underscores the ever-evolving role of β-glucans in shaping the future of food product development and innovation.

## 6. Advances in Bioavailability Studies of β-Glucan 

Bioavailability is a crucial parameter denoting the extent to which a consumed substance reaches its intended target, exerts its effects, and gets utilized. This metric is of paramount importance in pharmacology and nutritional science. The study of bioavailability in the context of soluble β-glucans has garnered relatively limited attention in the research community, making it a focal point for further investigation. In a study investigating the oral administration and gastrointestinal absorption of soluble β-glucans, it was revealed that the bioavailability of three distinct soluble β-glucans falls within a range of 0.5% to 4.9%. Among these, laminarin exhibited the highest bioavailability, followed by scleroglucan, while glucan phosphate displayed the lowest bioavailability at 0.5% [46]. A separate examination delved into the metabolism of barley β-glucans in the upper intestine using ileal effluents collected from an experimental diet containing barley. The findings were juxtaposed with in vitro β-glucan extraction procedures. In vitro, endogenous proteases solubilized approximately 28% of the β-glucan, which increased to 83% following non-starch polysaccharide (NSP) separation. Similarly, in the ileal effluent, around 60% of the β-glucan was solubilized. Importantly, the viscosity of the ileal effluent remained low, akin to a mucin benchmark. These results suggest that while β-glucan can be solubilized in the upper intestine, its viscosity may not significantly affect its bioavailability. 

Ref. [47] conducted a study investigating the metabolism and bioavailability of hydrogenated resistant glucan (HRG) and resistant glucan (RG) in both rats and humans. The study revealed that, unlike most high molecular weight carbohydrates that are partially digested and mildly fermented in healthy individuals, RG and HRG were subject to limited digestion and fermentation. Consequently, both RG and HRG demonstrated very low bioavailability in both human and rat subjects. This reduced bioavailability can be attributed to the high mobility of RG across intestinal bacteria, whereas the oligosaccharides present in pure RG exhibited limited mobility of their primary components. Furthermore, Ref. [48] investigated the impact of acetylation, using acetic anhydride at concentrations of 4–6%, on oat β-glucan. This study assessed alterations in the thermal, morphological, functional, and rheological properties of β-glucan concentrates with a 31% oat β-glucan content. Acetylation of the β-glucan molecule resulted in a reduction in its capacity to bind fat and an enhancement in glucose availability. Importantly, the increased bile acid binding capacity and greater swelling power were found to be essential for these functional improvements. Recent research using rat models suggests that the bioavailability of β-glucans falls in the 4–5% range, with soluble glucans capable of translocating from the gastrointestinal tract to the systemic circulation. While various gastrointestinal cell types, including mucosal dendritic and epithelial cells, are known to interact with β-glucans, the precise mechanisms underlying these interactions remain to be fully elucidated.

Current research reveals that β-glucan metabolism and bioavailability can vary based on their source and structure. Factors like enzymatic solubilization in the gastrointestinal tract and interactions with gut bacteria influence bioavailability. Additionally, acetylation is explored to modify β-glucans, impacting functionality and bioavailability. Future research areas include investigating mechanisms of absorption and metabolism, exploring source and structural differences, continuing research on functional modifications, and studying health implications. Comprehending β-glucan bioavailability is vital for optimizing its therapeutic potential, and ongoing research aims to fill knowledge gaps and enhance the use of β-glucans in various health-related applications.

## 7. Mechanistic Insights on Biofunctionalities of β-Glucan

### 7.1. Mechanism of Hypoglycemic Effect 

Diabetes, a chronic condition characterized by elevated blood sugar levels, serves as a precursor to various cardiovascular ailments, including hypertension, coronary artery disease, peripheral vascular disease, and atherosclerosis. According to the World Health Organization (WHO), the global prevalence of type II diabetes is surging at an alarming rate. The multifaceted etiology of diabetes encompasses factors such as dysfunctional β-cells, insulin resistance, aberrant mitochondrial activity, and apoptosis [49]. Notably, β-glucan emerges as a pivotal agent in mitigating insulin activity and postprandial blood glucose levels.

Researchers have been actively investigating the potential health benefits of β-glucans derived from cereals, primarily barley and oats. These compounds have garnered significant attention due to their ability to lower blood sugar and cholesterol levels and modulate insulin responses. Barley stands out as a low-glycemic-index food option compared to other cereals, making it a valuable dietary choice. Remarkably, the addition of just 4 g of barley-derived β-glucan to chapattis reduced the glycemic index from 54 to 30 [50]. The mechanism behind these effects involves β-glucan acting as a DF, delaying gastric emptying, and consequently slowing the release of glucose, ultimately leading to reduced blood glucose levels. Oat-derived β-glucan has also demonstrated the capacity to lower glycemia and insulin levels. Furthermore, it has been observed that oat β-glucan can hinder enzyme accessibility, resulting in decreased starch digestion and postprandial glycemia. Moreover, its gel-forming properties in the small intestine delay the interaction of digestive enzymes with nutrients and the subsequent absorption of glucose. This, in turn, leads to a temporary reduction in the peak postprandial blood glucose concentration. In addition to its glycemic control properties, β-glucan has been linked to the reduction of menaquinol, a form of vitamin K_2_ associated with type 2 diabetes. Studies have also shown that incorporating natural oat products enriched with β-glucans into the diets of individuals with diabetes can lead to improved glucose levels, particularly when consumed at higher daily doses. 

β-glucan, being a DF, escapes digestion in the small intestine and enters the large intestine, where it undergoes fermentation and produces short-chain fatty acids (SCFA). These SCFAs help maintain gut pH and regulate the secretion of gut hormones, playing a critical role in signaling satiety. Research conducted by [51] demonstrates the anti-diabetic potential of β-glucans, showing reduced cholesterol, triglycerides, and glucose levels in obese rats. Oat β-glucans were found to decrease the activity of key intestinal disaccharidases, lowering the glycemic response in a dose-dependent manner, both in vivo and in vitro, as these sugars are mixed with high-viscosity β-glucan gel [52]. This gel’s viscosity is inversely correlated with rises in post-meal blood glucose. Studies involving mice suggest that the effects of endogenous β-glucanases on hyperglycemia and LDL cholesterol management remain unaffected by the incomplete hydrolysis of barley β-glucans. Furthermore, the expression of the enzyme α-glycosidase is inhibited, and glucose transporters SGLT1 and GLUT2 are expressed less [52]. These factors not only regulate carbohydrate absorption but also elevate GLP-1 levels. Research reported a meta-analysis centered on measuring barley’s potency and its commodities in postprandial glycemia. The evaluation occurred for an entire set of 17 trials, comprising 68 distinct protocols and 212 individuals. This concluded that ingesting barley β-glucan reduced postprandial glycemic reaction. The reduction in postprandial glycemia was sufficiently significant to be considered scientifically meaningful [53].

β-glucan from cereals like barley and oats hold significant promise in managing blood sugar and cholesterol levels and improving insulin responses through various mechanisms, making them a topic of considerable scientific interest and potential clinical relevance. Exploring personalized β-glucan interventions, elucidating specific molecular pathways, and developing innovative β-glucan-enriched food products to optimize glycemic control, cholesterol management, and metabolic health in individuals with diverse dietary and genetic profiles would be an interesting future avenue.

### 7.2. Mechanism of the Cholesterol Lowering Effect 

Dyslipidemia, a chronic condition characterized by elevated lipid and cholesterol levels in the bloodstream, poses a significant risk for the development of life-threatening diseases such as myocardial infarction, ischemic and hemorrhagic stroke, and atherosclerotic vascular disease. It is imperative to investigate potential treatments to mitigate the rising mortality associated with dyslipidemia. The American Heart Association reported that 11.7% of individuals aged 20 or older, totaling 28.5 million people, have blood total cholesterol levels exceeding 240 mg/dL [54]. β-glucan emerges as a promising therapeutic agent, acting through multiple mechanisms to combat dyslipidemia. These mechanisms include reducing blood cholesterol levels, slowing bowel transit time, preventing constipation, increasing SCFA production, enhancing the growth of beneficial gut microbes, and reducing the risk of colorectal cancer.

One pivotal action of β-glucan is its ability to bind with bile acids, facilitating their excretion. This, in turn, reduces plasma cholesterol and bile acid levels. An increased elimination of bile acids promotes cholesterol metabolism in the liver, lowering lipid absorption and decreasing plasma cholesterol levels. Moreover, the fermentation of β-glucan in the large intestine produces acetate and butyrate, which inhibit cholesterol biosynthesis. β-glucan also participates in the degradation of LDL cholesterol. Recent research demonstrates that a daily intake of 3 g of β-glucan effectively lowers LDL cholesterol without significantly affecting high-density lipoproteins [55]. Furthermore, the high viscosity of β-glucan substantially impacts the absorption of cholesterol, fats, and other biomolecules in the gastrointestinal tract by increasing its viscosity. Incorporating fiber, particularly β-glucan-rich sources, into the diet offers a dual benefit: lowering serum cholesterol and reducing the intake of total fat, saturated fat, and dietary cholesterol [56]. 

The FDA has granted approval for the relationship between reduced blood cholesterol levels and increased DF absorption, specifically concerning psyllium and oat fiber. Notably, oat β-glucan supplementation has demonstrated a remarkable reduction in total cholesterol and LDL cholesterol by approximately 10% and 15% in a clinical trial involving slightly hypercholesterolemic individuals [57]. Further insights from studies involving oat-based diets in both rodents and humans suggest a link between increased acetate and butyrate levels and reduced cholesterol biosynthesis. Similarly, a comprehensive meta-analysis of 14 clinical studies reports a significant reduction in LDL cholesterol using barley β-glucan [58]. To delve into the mechanistic details, Ref. [59] explored hypercholesterolemic hamsters and observed that β-glucan from hull-less barley modulated the expression of key enzymes such as cholesterol 7-α hydroxylase (CYP7A1) and 3-hydroxy-3-methyl glutaryl-coenzyme A (HMG-CoA) reductase. This led to increased elimination of fecal lipids, resulting in decreased LDL cholesterol levels in the bloodstream. In vitro studies emphasize the significant impact of highland barley’s soluble DF in slowing cholesterol uptake, demonstrating a dose-dependent effect [60]. Human research, as exemplified by [61], indicates that the consumption of HB sprouts can enhance lipid metabolism and reduce the risk of cardiovascular disease, particularly in individuals with marginal cholesterol levels. In murine studies, the prolonged administration of highland barley β-glucan resulted in decreased serum total cholesterol, non-HDL cholesterol, LDL cholesterol, Lee’s index, and increased levels of SCFA. Similarly, mice subjected to higher doses of whole highland barley β-glucan exhibited reduced liver organ indices, abdominal fat, and liver lipid levels [62]. 

Notably, the molar mass of barley β-glucan plays a crucial role, with lower molar mass showing a 13% decrease in LDL cholesterol at a dosage of 5 g/day, while higher molar mass displays a 15% reduction [63]. An interesting dosage-dependent effect was observed in beverages fortified with β-glucan; the 5 g dose effectively lowered LDL and total cholesterol, whereas the 10 g dose did not [64], highlighting the significance of higher molar masses due to the solubility of solutions. A study was conducted with 29 hypercholesterolemia (early stage) children aged 6-14 years. This experiment assessed the performance of a packaged cereal containing β-glucan, including a minimal saturated fat and cholesterol intake, for lowering LDL cholesterol levels in children. In the initial four weeks, the children were kept on a diet of 3 g/d of β-glucan, and for the next four weeks, the children were provided with a substitute. In participants who were at least 80% in accordance (n = 18), total and soluble DF levels rose extensively (26.7%, *p*~0.01 and 30.8%, *p*~0.02, respectively), while LDL sank to an average of 5.3% (*p*~0.03). Individuals with a BMI under the median (25.7 kg/m^2^) had the greatest decrease in LDL levels(9.2%, *p*< 0.001) [65]. The β-glucan effects on the lipid profile, glycemia, and intestinal health (BELT) study looked into how 3 g of oat β-glucan per day affected plasma lipids, glucose levels in the fasting state, and intestinal sanity. The study took place in an 8 week, double-blind, placebo-controlled, cross-over randomized clinical experiment that enrolled 83 Italian adults who followed a Mediterranean diet and had mild hypercholesterolemia and narrow cardiovascular disease prospects. The BELT research shows an intermediate effectiveness of 3 g/day β-glucan supplementation in lowering LDL, total cholesterol, and non-HDL levels in moderate hypercholesterolemic patients despite being a Mediterranean culture [66].

Barley β-glucans of Mw, 290 kDa and 1350 kDa, at two distinct dosages, 3 and 5 g, were the subject of a pair of studies that examined the consequences of their use. When β-glucans were added to ready-to-eat cereal and fruit juice in the first research, both high and low MW were effective at lowering LDL and total cholesterol after six weeks of use in comparison to the control products without β-glucans. The second trial offered participants a breakfast of β-glucan-infused crepes, tortillas, oatmeal, or chips for five weeks. A substantial decrease in circulating total cholesterol was seen only with high β-glucan products, instead of the control goods made with wheat and rice. They concluded that the cholesterol-lowering benefits of β-glucans are determined by their physicochemical features (i.e., Mw) rather than their daily consumption [67]. Ref. [68] conducted a study where β-glucans were incorporated in a variety of dietary products, resulting in a noticeable reduction of LDL and TC. Another study that looked at administering 5 or 10 g of β-glucans to a beverage to supplement the typical diet for eight weeks found that β-glucans from oats instead of barley drastically dropped total cholesterol concentrations [64]. 

Research exploring the beneficial effects of β-glucans from various sources in dyslipidemia management is in its nascent stages, often involving small-scale clinical trials. The transition to large-scale trials for validation is essential. While the potential of β-glucans in dyslipidemia management is promising, significant questions persist. Detailed investigation into the influence of β-glucan properties, optimal dosage levels, and interactions with other dietary components is imperative. This knowledge will enable more precise and efficacious interventions for cholesterol management, ultimately reducing the risk of cardiovascular disease.

### 7.3. Mechanism of Immunomodulatory Effect of β-Glucan

The immune system is our body’s defense mechanism against invading pathogens and diseases. It comprises two primary components: the innate immune system and the adaptive immune system. The innate immune system acts rapidly and non-specifically against pathogens and includes cells like monocytes (macrophages), mast cells, dendritic cells, and complement proteins. These cells respond swiftly through mechanisms like phagocytosis, opsonization, and the release of cytokines, aiding in the differentiation of lymphocytes. On the other hand, the adaptive immune system, involving B and T lymphocytes and Natural Killer (NK) cells, provides a highly specific and long-lasting immune memory. B cells contribute to humoral immunity by producing antigen-specific antibodies, while T cells induce cytotoxic responses by secreting cytokines and chemokines.

A critical component in the realm of immunomodulation is β-glucan, a polysaccharide found in various sources, including cereals, fungi, and bacterial cells. This remarkable compound has the ability to serve as an adjuvant, generating immune signals. Its immunomodulatory effects are due to its recognition as a pathogen-associated molecular pattern (PAMP) by cell surface receptors known as pattern recognition receptors (PRRs). The innate immune system is the first line of defense, where β-glucan primarily exerts its influence. One key PRR that interacts with β-glucan is Dectin-1, which is expressed on myeloid cells like neutrophils, macrophages, and dendritic cells. Dectin-1 possesses an extracellular C-type lectin-like carbohydrate-recognizing domain (CRD), a transmembrane domain, and a cytosolic intracellular immune receptor tyrosine-based activation motif (ITAM). It has been demonstrated that Dectin-1 specifically recognizes a particular structural configuration of glucans, particularly β-glucans, with a minimum of seven glucan monomers in the backbone and one side chain branch. This recognition triggers downstream signaling that results in immune responses such as phagocytosis, respiratory burst, microbial cell destruction, and the secretion of cytokines [69].

Another receptor that interacts with β-glucan is Complement Receptor 3 (CR3), found on neutrophils, NK cells, and monocytes. CR3 plays a crucial role in the phagocytosis and opsonization of tumor cells through the binding of inactivated complement protein (iC3b). To activate CR3, β-glucan must bind to both β-1,3 glucan and iC3b. This interaction recruits NK cells and other phagocytic cells to the site of inflammation, stimulating phagocytosis and degranulation of opsonized complexes with iC3b, ultimately leading to the death of tumor cells. Toll-like receptors (TLRs) are another group of PRRs that recognize and bind to pathogen-associated molecular patterns (PAMPs). TLRs play a crucial role in activating both the innate and adaptive immune systems. Lactosylceramide, a glycosphingolipid found on the plasma membrane of immune cells, binds to β-glucan, triggering an immune response by inducing respiratory burst and secreting cytokines. β-glucan influences innate immunity and shapes the balance between T Helper (Th) 1 and Th2 cells. Maintaining this balance is essential, as an imbalance can lead to allergies and autoimmune diseases. β-glucan plays a role in shifting this equilibrium towards Th1 cells, reducing the risk of allergic diseases by altering the Th1/Th2 balance [70]. A study examined the impact of brewers’ yeast (1,3)-(1,6)- β-D-glucan ingestion on the frequency of common cold events in normal individuals. The current investigation showed that yeast β-D-glucan preparation improved the body’s ability to resist attacking bacterial and viral infections [71]. Grifola β–glucan can stimulate the growth of NK cells and lymphocytes in immunocompromised mice treated with cyclophosphamide (CTX). Grifola β–glucan may help safeguard mice from reduced immunity and bone marrow degradation resulting from cyclophosphamide by increasing the activity of kinases and transcription factors like p-Jak2/Jak2, p-Stat3/Stat3, and Socs3) in the Jak2/Stat3/Socs signaling cascade [72]. Another research sought to examine the influenced-by-time implications of oat beta-glucans affecting colon apoptosis and autophagy in the CD (Crohn’s disease) rat. Daily consumption of (high-molar-mass oat β-glucans) βGl and (high-molar-mass oat β-glucans) βGh dramatically decreased colitis through the time-varying alteration of autophagy and apoptosis, with βGl having a larger impact in apoptosis and βGh over autophagy. The pathway followed could possibly be explained by the responsiveness of receptors like Dectin-1 and TLRs [73]. Taken together, β-glucan’s immunomodulatory effects are multifaceted and intricate, impacting various immune system components. Through interactions with PRRs, such as Dectin-1, CR3, and TLRs, β-glucan initiates a cascade of signaling events that activate immune cells and the secretion of various cytokines and chemokines. This intricate network of interactions and signaling pathways ultimately leads to enhanced immune responses, making β-glucan a promising agent for immune modulation and disease prevention. Future research should focus on optimizing the use of β-glucan as an immunomodulatory agent, exploring its potential in treating autoimmune diseases, and uncovering its interactions with other immune components. Additionally, more studies are needed to understand the specific mechanisms involved in β-glucan’s immunomodulation. 

### 7.4. As a Potential Anticancer Molecule 

Cancer, a highly intricate and multifaceted group of diseases characterized by uncontrolled cellular proliferation, poses formidable challenges in the realm of treatment. It stands as the second leading cause of mortality worldwide, responsible for approximately one in every six deaths [74]. Annually, there are nearly 19.3 million reported new cancer cases, resulting in approximately 10 million global fatalities [75]. The current gold-standard therapies, including surgery, chemotherapy, and radiotherapy, are associated with considerable side effects and yield a less-than-ideal prognosis.

β-glucan exerts potent anticarcinogenic effects by stimulating the host’s immune system, thereby thwarting oncogenesis and inhibiting tumor metastasis. Its anticancer impact stems from a multi-faceted approach involving direct tumor inhibition, immune system enhancement, and inherent anticancer properties. Various factors influence its antitumor efficacy, including the source, molecular structure, branching pattern, and chemical modifications of β-glucan. The remarkable repetitive structure of β-glucans enables them to bind to specific receptors on the membranes of immune cells, eliciting a robust anti-tumor response. This binding activates innate immunity, leading to accelerated antigen presentation, increased production of reactive oxygen species (ROS), enhanced phagocytosis, and the secretion of critical cytokines [76]. The secreted cytokines play a pivotal role in activating both B cells and T cells, thereby initiating adaptive immunity through humoral and cell-mediated immune responses, respectively. 

To maximize the effectiveness of β-glucan’s anticarcinogenic properties, precise delivery throughout the body and the immune system is essential, underscoring the importance of understanding β-glucan trafficking [77]. Predominantly, oral administration serves as the primary mode of β-glucan delivery, although intraperitoneal (IP) and intravenous (IV) administration are also employed, albeit less frequently [77]. Following oral administration, β-glucans enter the proximal small intestine, where they are phagocytosed by intestinal epithelial cells or pinocytic microfold cells (M-cells). Subsequently, β-glucan is transported from the intestinal lumen to immune cells in Peyer’s patches. Upon encountering β-glucan, gastrointestinal macrophages migrate to the lymphatic system via the bloodstream [78]. In lymph nodes, β-glucan activates dendritic cells, which capture and imprison damaged tumor cells in the tumor microenvironment. This process leads to the differentiation and activation of antigen-specific CD4+ and CD8+ T-cells. Degraded fragments of β-glucan further activate neutrophils by binding with CR3 and modulating hematopoietic myeloid progenitors in the bone marrow. These events collectively trigger CD3-dependent cellular cytotoxicity (CR3-DCC) in proximity to opsonized iC3b-coated carcinogenic cells [79]. Orally administered β-glucan not only accelerates respiratory bursts and the rate of phagocytosis but also enhances the secretion of crucial cytokines, including interleukin-1 (IL-1), IL-6, and TNF-α within macrophages. Additionally, it regulates the acute phase of humoral immunity and augments the activity of lysozyme and ceruloplasmin in animal models [80]. These multifaceted mechanisms underpin the promising potential of β-glucan in combating cancer and stimulating immune responses.

The tumor microenvironment (TME) is an intricate network of elements, including blood vessels, extracellular matrix, malignant cells, and non-malignant immune cells. β-glucan, derived from fungi, possesses the remarkable ability to modulate the TME by regulating both innate and adaptive immune signals. This modulation begins with the activation of the host’s innate immunity through the binding of β-glucan to PRRs, triggering the differentiation, activation, and deployment of specific acquired adaptive immune cells, crucial for the host’s defense mechanism [81]. Macrophages, particularly those within the TME, significantly influence cancer prognosis. Notably, β-glucan’s interaction with Dectin-1 on tumor-associated macrophages (TAMs) accelerates the transition of M1 macrophages, enhancing antigen presentation and Th1 cytokine secretion [82]. Furthermore, β-glucan extracts and ganoderic acid from *G. lucidum* exhibit direct cytotoxic properties by activating M1 macrophages and subsequently destroying HepG2 cells [83]. The progression of the tumor is also influenced by Myeloid-derived suppressor cells (MDSCs), a distinct class of immune cells. Yeast-derived whole β-glucan particles (WGP) lead to a reduction in polymorphonuclear-MDSCs (PMN-MDSCs) through enhanced respiratory burst and apoptosis. β-glucan also impacts B lymphocyte differentiation and activation, promoting the development of humoral immune responses via the secretion of cytokines such as IL-6, IL-8, and TNF-α, mediated by the activation of NF-κB and AP-1 [84]. However, tumor cells can limit dendritic cell antigen presentation by secreting IL-10 and vascular endothelial growth factor (VEGF), evading the immune response [85]. Astragalus polysaccharide (APS) enhances dendritic cell activation by upregulating MHC-II, CD-80, and CD86 on dendritic cell surfaces, promoting synergistic interactions between T cells and dendritic cells [86]. Moreover, the TME orchestrates a process to stimulate angiogenesis to enhance oxygen and nutrient supply and eliminate metabolic waste, countering the hypoxic and acidic microenvironment. Furthermore, sulfated derivatives of glucan from *Phellinus ribis* (PRP-S1 and PRP-S2) inhibit tumor angiogenesis by reducing VEGF expression [87]. In an experiment, 20 participants who had terminal malignant tumors were administered a β-(1,3)/(1,6) D-glucan formulation and evaluated for tolerance and impact on hematopoiesis during chemotherapy. The findings suggest that β-glucan may improve hematopoiesis in cancer victims undergoing chemotherapy [88]. When paired with chemotherapy, Grifola β–glucan reduced the dimensions of breast, lung, and liver tumors in more than sixty percent of the subjects in contrast to chemotherapy isolated [89]. Research conducted revealed that oat β-D-glucan is able to hinder HTB-140 melanoma cells by escalating the stimulation of caspase-3/7 along with the surge of phosphatidylserine towards the extracellular face of the plasma membrane, which suggests the ordination of mitochondrial apoptosis [90]. A low-MW oat β-glucan showed firm activity of caspase-12 against A431 and Me45 cancer cell lines, leading to apoptosis and thereby showing its potential anticancerous activity [91].

In summary, β-glucan holds immense promise in the fight against cancer by stimulating the immune system, inhibiting tumor growth, and modulating the TME. However, there remain gaps in our understanding of the optimal delivery methods and the precise mechanisms underlying β-glucan’s multifaceted actions within the TME. Future research should focus on refining the delivery strategies, elucidating the specific immune pathways involved, and exploring potential combination therapies to maximize β-glucan’s anticancer potential.

### 7.5. Apoptosis 

Apoptosis, a vital process in multicellular organism development, is a regulated form of cell death. It primarily eliminates damaged cells with irreversible DNA damage, contributing to the organism’s overall health. Key apoptotic hallmarks include chromatin condensation, cell shrinkage, membrane blebbing, and the loss of contact inhibition. Two primary pathways, the extrinsic and intrinsic pathways, orchestrate apoptosis. The extrinsic pathway relies on external signals, like TNF family death receptors (e.g., FasL/FasR), which activate caspase-8 and caspase-3, ultimately causing cell death [92]. In contrast, the intrinsic pathway involves factors such as the Bcl-2 protein family, with anti-apoptotic (e.g., Bcl-2) and pro-apoptotic (e.g., BAX) members. Dysregulation of these proteins can lead to cancer.

Various studies have elucidated the potent mechanisms by which different β-glucan isoforms or crude extracts can stimulate apoptosis, offering promising avenues for anti-cancer interventions. Notably, β-glucans sourced from bacterial origins have demonstrated their ability to induce apoptosis in SNU-C4 cells, as confirmed by TUNEL assay validation. This pro-apoptotic effect is underpinned by the upregulation of caspase-3, Bax, and the downregulation of Bcl-2. Furthermore, these β-glucans induce dose-dependent changes in cell morphology, the formation of apoptotic bodies, and chromatin condensation [93]. Hot water extracts from Chaga mushrooms have been shown to elevate apoptosis in HT-29 colon cancer cells. This effect is attributed to the modulation of key apoptotic regulators, including increased Bax and caspase-3 levels, coupled with a reduction in Bcl-2 expression [94]. In another line of investigation, β-glucans derived from *Agaricus blazei Murill* have exhibited a multifaceted mechanism of apoptosis induction. This includes the acceleration of cytochrome-C release, enhanced p38 MAPK activity, Bax translocation to mitochondria, and the activation of caspase-9. These molecular events culminate in apoptosis induction and contribute to reduced metastasis in a mouse tumor model [95]. Studies with extracts from *Inonotus obliquus* have unveiled their inhibitory effect on B16-F10 melanoma cells and human hepatoma HepG2 cells. This inhibition is associated with G0/G1 cell cycle arrest, apoptosis induction, and a reduction in key regulatory proteins like p53, p27, pRB, cyclin, and CDK [96]. Notably, extracts from *Ganoderma lucidum* have exhibited the ability to enhance apoptosis in ovarian cancer cells. This effect is achieved through the modulation of Akt, p53, and caspase-3 activation [97]. Breast cancer cells expressing estrogen receptors have been effectively hindered from proliferation through the treatment with β-1,3 glucan from *Lentinus edodes* (LNT). This anti-proliferative effect is corroborated using Western blotting, which revealed elevated p53 levels, phosphorylated ERK1/2, PARP-1, caspase-3, and reduced levels of p65, NF-kB, TERT, and MDM2 in tumor cells [98]. Furthermore, oat β-glucan has demonstrated its potential to induce apoptosis in human skin melanoma HTB-140 cells in a concentration-dependent manner. This action is marked by enhanced caspase 3/7 activation and phosphatidylserine accumulation on the cell surface, both of which drive apoptosis [90]. Lastly, extracellular β-glucans isolated from botryosphaeran and lasiodiplodan have been shown to induce oxidative stress in MCF-7 cells. The underlying mechanism of β-glucan-regulated apoptosis is supported by RT-PCR results, indicating the increased mRNA expression of forkhead transcription factor (FOXO-3a), p27, p53, AMP-activated protein kinase (AMPK), and a concomitant decrease in p70S6K [99].

**Figure 5:**
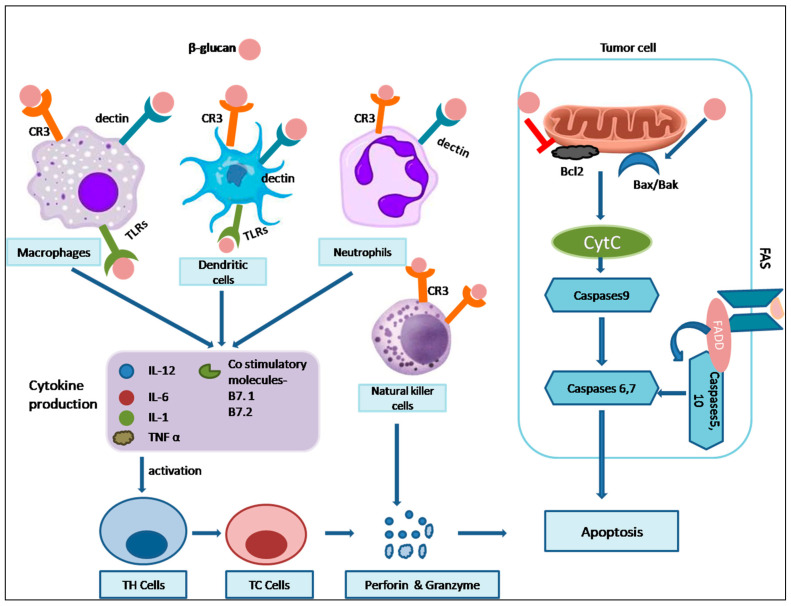
Immunomodulatory and proapoptotic effects of β-Glucan. β-glucan binds to the receptors such as dectin-1, CR-3, and TLRs present in immune cells like macrophages, dendritic cells, neutrophils, and natural killer cells to induce the production of cytokines and co-stimulatory molecules, including IL-12, IL-6, IL-1, TNF-α, B7.1, and B7.1 which results in the activation of Th and Tc cells. TH cells and Tc cells, along with natural killer cells, release perforin and granzyme, resulting in the apoptosis of cells. Also, β-glucan in the tumor cells binds to the FAS receptor, promotes Bax/Bak, and inhibits Bcl2, leading to the activation of caspases and, consequently, apoptosis of tumor cells. Abbreviations: TLRs: Toll-like receptors, CR3: compliment receptor 3, IL: interleukin, TNF-α: tumor necrosis factor-α, TH: T helper cells, TC: T cytotoxic cells, Bcl2: B-cell lymphoma, Bax: Bcl associated X-protein, Bak: Bcl-2 antagonistic/killer, CytC: Cytochrome C, FAS: TNF Family death receptor, FADD: FAS associated protein and death domain.

The highlighted studies shed light on the multifaceted mechanisms by which various β-glucan isoforms and extracts induce apoptosis, holding significant promise for potential anti-cancer interventions. These mechanisms involve the modulation of key apoptotic regulators, the upregulation of pro-apoptotic factors like caspase-3 and Bax, and the downregulation of anti-apoptotic elements such as Bcl-2, as illustrated in Figure 5. The intrinsic and extrinsic pathways of apoptosis are influenced, and various cancer cell types are targeted, offering diverse strategies for combatting cancer. However, there are notable gaps and future perspectives in this area of research. While the mechanisms of action are becoming clearer, further studies are needed to unravel the specific signaling pathways and molecular interactions that underlie the effects of β-glucans on apoptosis. Additionally, more comprehensive in vivo studies are necessary to validate the potential clinical applications of these findings. The development of standardized β-glucan formulations and dosage regimens is also critical in translating this research into practical cancer therapies. Moreover, investigating the interplay between β-glucans and existing cancer treatments and exploring their potential synergy or antagonism is an important avenue for future research. Lastly, the safety profiles and long-term effects of β-glucans in human trials warrant careful examination. Overall, continued research in this field promises to expand our understanding of apoptosis regulation and improve cancer treatment strategies.

### 7.6. β-Glucan in Gut Microbiota

The gut microbiota, often referred to as the “second brain” of the body, plays a pivotal role in maintaining overall human health. One of the critical functions of gut microbiota is the utilization of β-glucan, a DF, as a substrate. This fiber is essential for promoting healthy biological functions. Any disruption in the composition of gut microbes can lead to increased intestinal permeability, causing colonic inflammation that may contribute to the development of colon cancer. The human gut is home to an astounding population of over 100 trillion microbial organisms, collectively encoding a far greater number of genes than the human system itself. These microorganisms are indispensable for sustaining a healthy gut environment, actively participating in processes such as angiogenesis, modulation of signaling cascades, vitamin K biosynthesis, and the regulation of metabolic pathways [100].

The introduction of indigestible polysaccharides from various sources into the diet can enhance health by fostering the growth of beneficial microbial communities [101]. Once they escape digestion, these DFs reach the large intestine, where they undergo fermentation, producing SCFA, including acetate, propionate, and butyrate. Furthermore, the fermentation process yields compounds like indole, tryptophan derivatives, and secondary bile acids, all of which contribute to the advantageous effects observed [102]. In the processing of β-(1,3)-glucans, gut microbiota employs enzymes from the glycoside hydrolase family, such as β-(1,3) glucosidase (EC 3.2.1.58) and β-(1,3) glucanases (EC 3.2.1.6 and EC 3.2.1.39). β-(1,3)-glucanases break down internal glucosidic linkages, resulting in oligopolymers, while β-(1,3)-glucosidases act on the linkage at the non-reducing ends, releasing free glucose monomers [103]). The presence of carbohydrate-binding domains on a fraction of endo-acting β-(1,3)-glucanases enhances their binding capacity to water-insoluble compounds. To break down β-(1,6)-linked branched chains, another crucial enzyme, β-(1,6)-glucanase (EC 3.2.1.75), belonging to the GH30 family, is essential. The proliferation of epithelial cells may alter gut permeability and lead to inflammation, significantly affecting overall human health. *Lactobacilli* and *Bifidobacteria* species are particularly beneficial for gut health, and β-glucans from sources like oats and barley have been found to promote the growth of these species. [104] studied the effects of orally administered β-glucan on rabbit gut health and body growth, revealing improvements in body weight, total feed consumption, and antioxidant enzyme activity, indicating a modulation of anti-inflammatory responses and improved gut health. Furthermore, research involving the continuous administration of β-glucans from cereals to rats demonstrated an increase in the numbers of *Lactobacillus* and *Bifidobacterium* after 3, 6, and 7 weeks [105]. A clinical investigation evaluated the prebiotic ability of barley β-glucan in vivo using a randomized, double-blind, placebo-controlled design. Fifty-two healthy participants aged 39–70 had been allocated to have a cake with 0.75 g of barley β-glucan or a placebo every day for 30 days. The study found that taking a daily cake with barley β-glucan was tolerated effectively and had strong bifidogenic characteristics in healthy seniors [106].

The exploration of gut microbiota and its interaction with dietary β-glucan opens up several compelling avenues for future research. Understanding the specific enzymes and metabolic pathways that govern β-glucan processing by gut microbes is a fundamental research gap. This knowledge could optimize the dietary delivery of β-glucan for therapeutic purposes. Furthermore, investigating how gut microbiota composition can prevent colonic inflammation and related diseases, such as colon cancer, is a promising area. Identifying the key microorganisms and their functions in maintaining gut health is crucial for targeted interventions. The potential for DF to modulate the gut microbiota is an exciting research prospect, offering insights into how different fibers influence microbial communities and their metabolites, including SCFAs. Long-term studies on the effects of β-glucan supplementation in populations with metabolic syndrome are necessary to determine sustained benefits and potential side effects. The complex relationship between gut microbiota, β-glucan, and human health presents numerous research opportunities. Investigating microbial interactions, their impact on diseases, and long-term dietary interventions can enhance our understanding of gut health and personalized nutrition strategies.

## 8. Industrial Application of β-Glucan in Food, Medical and Cosmetics

**Figure 6:**
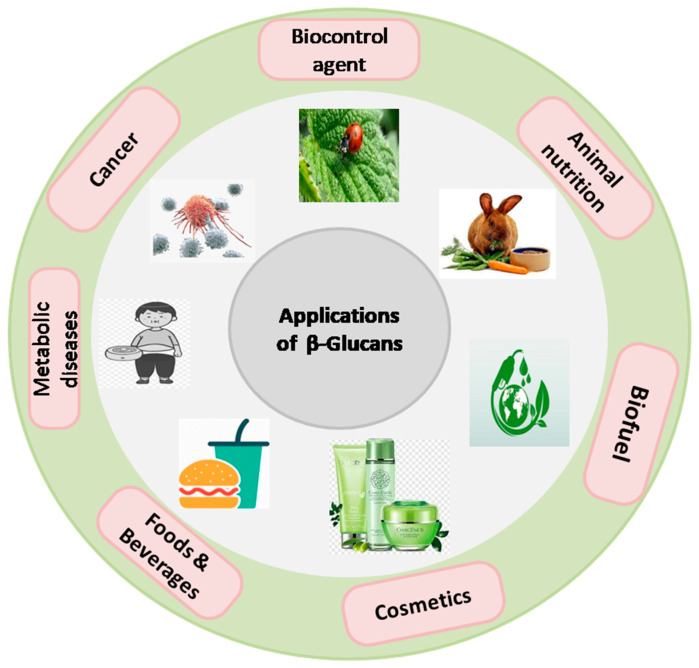
Multifaceted applications of β-glucan in different industries.

In recent years, β-glucan has garnered significant attention due to its diverse applications within the industrial sectors of food, medicine, and cosmetics (Figure 6). Within the realm of food science, β-glucan has emerged as a functional ingredient, contributing to the enhancement of product textural properties, shelf stability, and nutritional profiles. Its remarkable immunomodulatory attributes have found relevance in the medical domain, potentially offering therapeutic avenues for an array of health conditions, including cardiovascular ailments and diabetes. Furthermore, in the cosmetic industry, β-glucan has been harnessed for its innate capacity to soothe and moisturize the skin, presenting a natural and efficacious component for skincare formulations. The multifaceted and dynamic utility of β-glucan underscores its significance as a valuable resource, fostering innovation and progress across these diverse industrial sectors. Further elaboration on recent advancements in each sector is presented in subsequent sections.

### 8.1. Food and Beverage Industries

β-glucan, a multifunctional polysaccharide, is a pivotal ingredient in the food and beverage industry due to its remarkable thickening and gelation properties and ability to enhance solution solubility. This versatile compound augments the flavor, texture, and appearance of culinary delights such as ice creams, gravies, and salad dressings and functions as a calorie-reducing fat mimic to meet the demand for healthier food options. Despite its promising utility, β-glucan’s flow behavior and gelling characteristics pose significant technical challenges. These challenges manifest as limited yield, precipitation issues during the storage of beer, and delayed filtration of solutions or slurries in industrial applications. To address these concerns, there is a growing interest in technologically advanced β-glucan texture enhancers, which have the potential to revolutionize their incorporation into various food matrices. Recent research, as exemplified by [42], highlights the potential of hull-less barley β-glucan as an effective thickener and DF source in beverages and liquid products, backed by comprehensive rheological evaluations. However, the formidable viscosity of β-glucans and their complex handling characteristics in industrial processes have thus far constrained their widespread adoption. Therefore, it is imperative to consider the intricate interplay between β-glucan concentration and the resultant properties it imparts to finished products. [43] underscore how the incorporation of barley components into foods significantly enhances water absorption, consequently influencing the viscoelastic attributes of the final products.

β-glucan serves as a proficient thickening agent, fat substitute, emulsifier, and stabilizer for foams and emulsions. When combined with other hydrocolloids, it becomes a valuable source of soluble fiber in meat emulsions. Furthermore, β-glucan demonstrates potential as a prebiotic in high-folate products. Recent research by [107] delves into the post-prandial metabolic effects of functional bread enriched with β-glucans and resistant starch. This study assessed glycemic and insulin levels, ghrelin, GLP-1, and PYY responses, as well as individual assessments of hunger and sensory attributes. Incorporating dates and *Agaricus bisporus* mushrooms into bread recipes will enhance the protein and iron content, nutritional quality, texture, sensory acceptance, and shelf life [108]. For the dairy sector, the addition of high molecular weight oat β-glucan to milk yields products with reduced calories and cholesterol. The phase behavior, rheological characteristics, and microstructure of these dairy products impact their flow behavior, especially at higher concentrations [109]. Yogurts enriched with pectin and β-glucan exhibit enhanced proteolysis, reduced release of large peptides, and a higher proportion of free amino acids compared to counterparts with starch or no β-glucan [110]. The β-glucan content in food products, whether extracted from commercial sources or laboratories, varies significantly, with food matrix properties influencing the ideal β-glucan concentration. For instance, noodles typically contain up to 10% β-glucan, while soups may have around 2%, and meat emulsions range from 0.3% to 3%. Milk and dairy products contain about 2.5%, and bread can incorporate between 5.5% and 20%. A study by [111] investigated the influence of hydroxypropyl methylcellulose, yeast β-glucan, and whey protein isolate on gluten-free bread made from rice starch-based formulas. The sensory evaluation revealed that yeast β-glucan-enhanced rice starch bread was well-received. The optimal β-glucan levels for various food groups remain unclear, but electrostatic interactions between proteins and β-glucan during aggregation have been established [112]. Additionally, β-glucans, while improving dough production due to their hydrocolloid nature, can affect the structural qualities of baked goods, leading to increased hardness, gumminess, and decreased cohesion and elasticity. Despite sensory differences, consumers generally favor bread made with wheat flour over hulled barley whole grain flour (HLB WGF). Nonetheless, these findings provide valuable insights for the formulation of functional bakery products [113].

Recent studies, including those by [114], have mainly focused on β-glucan as an encapsulating agent for substances such as fish oil and probiotics. As we enter the twenty-first century, consumer demand is driving food processors to seek natural ingredients over synthetic ones, making DF and β-glucan promising candidates in the growing nutraceutical market [115]. β-glucan, derived from barley and oats, was incorporated into two different soups. Freezing, as predicted, significantly impacted the molecular weight and sensory attributes of these soups, exerting a profound influence on their quality [116]. Meanwhile, researchers successfully developed a barley extract beverage reminiscent of tea, with extraction temperatures ranging from 150 °C to 280 °C. The extracted materials were analyzed for physical and chemical properties, revealing 5-Hydroxymethyl-2-furaldehyde as a notable antioxidant [117]. Blending whey protein isolate with β-glucan yielded a high-quality, palatable beverage with an 18-week shelf life, albeit without prominent sweetness, sourness, or flavor intensity. Notably, oxidative cleavage driven by OH- radicals played a pivotal role in β-glucan degradation in these beverages. Understanding the thermodynamic properties of combined ingredients is crucial for innovating culinary applications and novel product development. When comparing beverages made with 0.5–0.7% β-glucan and an equal amount of pectin to those with 0.3% thickener, the former exhibited enhanced viscosity without a significant difference in consumer approval. Colorimetric analysis of these beverages showed an initial decline in color stability, particularly in those with higher β-glucan content. Additionally, cloud loss was observed during the first three weeks of storage in β-glucan-infused beverages. These findings underscore the potential of barley β-glucan to provide health, nutritional, and functional benefits crucial for product development within the food and beverage industry [118].

Despite the significant strides made in understanding the diverse applications of β-glucan in the food and beverage industry, several critical gaps and avenues for future research remain to be explored. First, a comprehensive investigation into the optimal β-glucan concentrations and their interactions within various food matrices is essential, as this could guide the development of tailored formulations for specific products, addressing sensory and textural issues while maximizing health benefits. Moreover, there is a need for further exploration of advanced processing techniques and technologies to overcome the challenges related to the formidable viscosity and complex handling characteristics of β-glucan in industrial applications. This could enhance the efficiency of incorporating β-glucan into food products and facilitate its widespread adoption. Additionally, while previous research has shown promise in using β-glucan as a fat mimic and calorie-reduction agent, more studies are needed to assess its long-term effects on consumer health and to optimize its functionality in this capacity. Furthermore, the potential prebiotic properties of β-glucan and its interactions with gut microbiota remain an intriguing area for investigation, as it could lead to the development of functional foods with enhanced digestive benefits. Finally, research into the structural modifications of baked goods and their sensory attributes when β-glucan is included can provide insights into the creation of palatable and healthy bakery products. These unexplored areas promise to further advance the applications of β-glucan in the dynamic food and beverage industry.

### 8.2. Cosmetic Industries

β-glucan has found widespread application in the cosmetics and personal care products industry. Scientific studies have revealed its multifaceted benefits in skincare. β-glucan, derived from sources such as mushrooms, cereals, and microorganisms, exhibits soothing, moisturizing, and anti-irritant properties. Its skin-regenerating capabilities are well-documented, making it a valuable addition to cosmetic formulations. Notably, β-glucan is lauded for its ability to hydrate the skin and mucosa, revitalizing them to their optimal condition. Furthermore, researchers have harnessed β-glucan’s exceptional moisture retention properties for innovative applications. In addition, the efficacy of natural β-glucan from various sources has been explored for improving overall skin health, as detailed in the work of [119]. β-glucan’s significance extends beyond skincare; it has been leveraged for addressing diverse concerns, including skin aging, vaccine production, eye ulcer treatment, and dermatological procedures. Its presence in cosmetic products, such as sunscreens, suspensions, creams, and powders, is underpinned by its role in stimulating collagen production, reducing fine wrinkles, and alleviating conditions like eczema. Furthermore, its wound-healing potential and capacity to mitigate oxidative tissue damage make it a promising candidate for wound dressing applications. The marriage of β-glucan with chitosan, as explored by [120], opens doors to intriguing therapeutic wound dressing possibilities. Research also suggests that β-glucan may play a role in reversing skin aging and minimizing wrinkles, demonstrating its versatility and appeal in the cosmetic industry. From mushroom-derived β-glucan’s potential as an eye drop to its ability to fortify the immune system and scavenge free radicals, it is evident that β-glucan’s applications are far-reaching. In addition, studies indicate that baker’s yeast-derived β-glucan has promising prospects in cosmetics and therapeutic products. To support these claims, experiments have shown that CM-glucan stimulates keratinocyte growth, enhances skin’s resilience against damage caused by detergents, and accelerates the regeneration of the stratum corneum. Oat β-glucan, such as “Avenacare,”is a favored ingredient in personal care and cosmetic products, celebrated for its calming, hydrating, and anti-irritant properties.

Despite the extensive body of research highlighting the diverse applications and benefits of β-glucan in the cosmetics and personal care industry, several potential research gaps and promising avenues for future exploration emerge. One key area that warrants further investigation is the optimization of β-glucan’s extraction methods, particularly from various natural sources. Enhanced methods for maximizing the yield and purity of β-glucan could further expand its utility in cosmetic formulations. Additionally, while there is evidence of its effectiveness in skin regeneration, there is room for more in-depth studies to elucidate the underlying mechanisms and precise dosages required for optimal results. The role of β-glucan in addressing specific skin conditions, such as eczema and skin aging, could be a focal point for future research, including clinical trials to establish its efficacy in real-world scenarios. Moreover, understanding the synergistic effects of β-glucan when combined with other skincare ingredients could lead to the development of more potent formulations. Research on the long-term effects of β-glucan in skincare products and its interaction with varying skin types and conditions is also essential. While the potential use of β-glucan in wound dressings is promising, further studies can delve into the development of innovative wound care products that leverage its properties effectively. Investigating the application of β-glucan in hair care products and its benefits for scalp health is another exciting avenue to explore. Rigorous safety and toxicity assessments are needed to provide a comprehensive scientific basis for the incorporation of β-glucan in cosmetics and personal care products. This includes investigating any potential side effects or sensitivities in different populations. By addressing these research gaps, we can unlock the full potential of β-glucan in enhancing skincare and personal care products.

### 8.3. Medical Industries

The significance of β-glucans in the medical industry cannot be overstated. Clinical trials dating back to the 1980s introduced the concept of using fungal β-glucans as adjuvant cancer therapies, opening new vistas for treatment approaches. In the ever-evolving landscape of medical research, one key player has emerged as a beacon of hope in the ongoing battle against cancer: β-glucans. These remarkable compounds, derived from various sources such as lentinan, D-fraction, and schizophyllan, have drawn considerable attention due to their pivotal role in revolutionizing cancer therapy [121]. As one of the leading causes of death globally, cancer continues to thwart conventional treatment strategies, including radiation, chemotherapy, and surgical resection. β-glucans, however, offer a transformative approach. These natural agents have demonstrated potent anticancer effects against a spectrum of malignancies, encompassing gastrointestinal, breast, and lung cancers. They act by inducing cytotoxicity in cancer cells, effectively inhibiting proliferation and bolstering apoptosis through intricate molecular pathways [122]. The profound potential of β-glucans in the realm of medical science offers a tantalizing glimpse of a future where cancer treatment is more effective, safer, and less prone to the horrors of metastasis and recurrence. This ongoing research heralds the dawn of a new era in cancer therapy, underlining the pivotal role of β-glucans as a beacon of hope and a catalyst for innovation in the medical industry.

Furthermore, the impact of β-glucans extends beyond the direct combat with cancer. Their immunomodulatory properties trigger a cascade of immune responses, including the secretion of pro-inflammatory and anti-inflammatory cytokines and the activation of NK cells, T cells, and macrophages, all of which actively engage with tumor cells [123]. Recent studies have probed the immunological underpinnings of various β-glucans, illuminating their capacity to enhance phagocytosis and IL-2 release and to restrain cancer growth. Additionally, these compounds have shown promise in ameliorating the effects of experimental infections [124]. Recent research has identified β-glucans as compounds with great potential for use in treatments for COVID-19, which is caused by coronavirus 2 known as SARS-CoV-2 [125]. β-glucans have also been reported to boost immunity and reduce COVID-19 symptoms as preventative supplements when taken orally [126]. They can stimulate several immune pathways, including the production of antibodies, and promote an immunomodulatory effect, improving the cellular pattern involved in the infectious process and decreasing the cytokines involved in the inflammatory course of COVID-19 [126]. The β-glucans from *Lentinula edodes* (lentinan) were recently studied and cited as molecules that act to reduce the lung damage from pneumonia caused by the SARS-CoV-2 virus because of its cytoprotective effect [125]. There have not been many studies on autoimmune disorders; however, an extract derived from *Agaricus blazei* was tested in inflammatory bowel diseases, with modest results on inflammatory cytokines or clinical symptoms [127]. Additionally, β-glucans accelerate wound healing by enhancing macrophage infiltration, encouraging tissue granulation, collagen synthesis, and skin re-epithelialization. Using soluble yeast β-1,3-glucan allowed 59% of all ulcers to heal by week 12 compared to 37% of the control group, according to another clinical trial. It is hypothesized that the gel-forming capabilities of β-glucans, which regulate bile acid and cholesterol metabolism, play a major role in mediating the cholesterol-lowering actions of β-glucans. By reducing the intestinal absorption of dietary cholesterol and blocking bile acid reabsorption, β-glucans also increase the need for bile acids to be synthesized from cholesterol catabolism, which lowers the LDL percentage of blood cholesterol. However, more recently, the regulation of cholesterol homeostasis has been linked to the effect of β-glucans activity on gut microbiota modulation, specifically on those bacterial species that influence bile acid metabolism and generation of short-chain fatty acids [128]. Oats or oat β-glucan consumption was linked in several randomized controlled studies and subsequent meta-analyses to reduced levels of LDL cholesterol as well as other improved markers of cardiovascular disease risk.

The multifaceted potential of β-glucans in the medical industry presents a rich landscape for future research endeavors. While significant progress has been made in understanding their role in cancer therapy, several notable research gaps remain. First, there is a need for further clinical trials and mechanistic studies to elucidate the optimal dosage, administration methods, and long-term effects of β-glucans in cancer treatment. Additionally, exploring their synergistic potential in combination with conventional therapies could enhance their clinical utility. Moreover, the immunomodulatory properties of β-glucans, especially in the context of infectious diseases like COVID-19, require continued investigation. Research could focus on how β-glucans interact with the immune system and their potential as prophylactic and therapeutic agents against a broader spectrum of viral infections. Further understanding their impact on autoimmune disorders and their mechanisms of action is another avenue for exploration. Wound healing, a vital aspect of medical care, could benefit from more comprehensive research into the application of β-glucans. Expanding clinical trials and elucidating the specific pathways through which β-glucans accelerate tissue repair and reduce ulcers are essential to harness their full potential in this field. In the realm of cholesterol management, ongoing research into the impact of β-glucans on gut microbiota and their role in regulating cholesterol homeostasis holds promise. More studies on the long-term effects of β-glucans on lipid profiles and their potential in preventing cardiovascular diseases are necessary. These research avenues are poised to unlock the full range of benefits that β-glucans can offer in the field of medical science.

## 9. β-Glucan’s Global Trade Potential

Barley and oats are well-recognized as prominent sources of plant-derived β-glucan, with barley boasting a higher β-glucan content than oats and yeast. This high β-glucan content, combined with an impressive extraction rate of up to 80%, makes barley an efficient and cost-effective choice for obtaining this vital DF. As the demand for natural, plant-based ingredients continues to rise, barley and oats are poised to meet the preferences of health-conscious consumers seeking natural products. This trend reflects the growing awareness of the health benefits associated with these grains, making them an attractive option for individuals looking to incorporate more plant-derived nutrients into their diets. Consequently, cereals, with a strong emphasis on barley and oats, are widely regarded as the preferred commercial sources of β-glucan. Commercial products in the market exhibit significant variations in the process of β-glucan isolation, concentration, molecular weight, water holding capacity, and viscosity. Researchers have undertaken various efforts to incorporate β-glucan into a diverse range of food products, including those based on cereals, meat, and dairy [129]. This underscores the multifaceted applications of β-glucan within the global market.

The global market for food-grade β-glucan is poised for significant growth. Current data from Market and Markets.com indicates that the majority of β-glucan manufacturing companies are concentrated in the United States (e.g., Super Β-glucan, Kemin Industries (North America, USA), Merck (New Jersey, USA), International Flavors & Fragrances Inc. (New York, USA)) and the European Union (e.g., Tate and Lyle PLC (London, UK), Kerry Group PLC (Tralee, County Kerry, Ireland), Ohly GmbH (Hamburg, Germany)) [130]. This distribution highlights the potential for market expansion in other global regions, emphasizing international market development prospects within the β-glucan industry. The β-glucan market is categorized into specific segments, including cereals and grains, yeast, mushrooms, and seaweed. The cereals and grains sector is anticipated to dominate, surpassing USD 229 million in 2023, and is projected to continue growing with a compound annual growth rate (CAGR) of 8.1%. This sector’s preeminence can be attributed to the long-standing tradition of extracting β-glucan from these sources due to their ease of production and processing. The current global market valuation for β-glucan, derived from various sources, is approximately USD 500 million. It is expected to experience robust growth, surpassing USD 700 million by 2028, with a CAGR of 7.9%. Specifically, the North American β-glucan market is forecasted to reach USD 272.7 million by 2028, exhibiting a CAGR of 8.3%, while the European Union is expected to achieve a market size of approximately USD 200 million by 2028, with a CAGR of 8.1%. The expansion of the Asia Pacific market is driven by health-conscious consumers and the abundant supply of cereals and grains, the primary source of β-glucan. The growth of the global β-glucan market is primarily driven by increasing consumer health consciousness and rising health-related expenditures. Moreover, the diverse applications of β-glucan across various industries contribute to market growth. Nevertheless, regulatory inconsistencies and price volatility in raw materials pose challenges to this growth. However, the market benefits from untapped applications for β-glucan, and the growing demand for natural and plant-based ingredients presents opportunities for further expansion.

## 10. Conclusions and Future Perspective

In conclusion, β-glucan, a complex polysaccharide found in various natural sources, including cereals, mushrooms, and algae, holds significant promise in nutrition, health, and industry. Recent advances in research have shed light on its structural characteristics, sources, bioavailability, and biosynthetic pathways, providing valuable insights into its potential applications. Here, we consolidate the key findings and provide future perspectives for the multifaceted field of β-glucan research. Recent research has unveiled crucial insights into the structural characteristics of β-glucan and the genetic underpinnings of its synthesis. Genes like CslF6 have been identified as essential for β-glucan production, enhancing our understanding of the biosynthetic processes involved. The dynamic nature of β-glucan levels during plant growth and development adds complexity to the field, necessitating further investigation. β-glucan’s diverse structural properties and functional attributes have significant implications for human health. Notably, it exhibits the potential to reduce cholesterol levels and offer immune modulation, making it a valuable dietary component. Research on its hypoglycemic effects and potential for weight management further underscores its relevance in the context of human health. The future of β-glucan research holds exciting prospects. Here, we have highlighted seven key areas of future exploration:

1. Genetic and epigenetic regulation: Understanding the regulatory mechanisms governing β-glucan production in plants at both the genetic and epigenetic levels will deepen our knowledge of plant biology. Advances in genome editing technologies, such as CRISPR/Cas9, offer the potential for precise engineering of β-glucan content and structure in crops, with implications for enhanced nutritional value and industrial applications.
2. Impact on plant growth and development: Exploring the effects of β-glucan manipulation on plant growth, development, and immunity will contribute to a broader understanding of plant biology and its applications in agriculture.
3. Bioavailability and gut interactions: Investigations into β-glucan bioavailability and its interactions with gut bacteria are needed to optimize its therapeutic potential. These insights will play a pivotal role in enhancing the health benefits associated with β-glucan consumption.
4. Personalized nutrition: Exploring personalized β-glucan interventions, elucidating specific molecular pathways, and developing innovative β-glucan-enriched food products can optimize its use for glycemic control, cholesterol management, and metabolic health tailored to individuals with diverse dietary and genetic profiles.
5. Clinical trials and medical applications: Further clinical trials are essential to validate the therapeutic potential of β-glucans in areas such as cancer treatment, immunomodulation, and managing metabolic syndrome. These trials will help translate research findings into practical treatments.
6. Sustainability and green chemistry: Integrating sustainable practices and green chemistry principles into β-glucan production and purification processes will enhance their environmental sustainability. This includes exploring innovative approaches like nanotechnology and advanced chromatographic techniques.
7. Advanced food and cosmetic applications: Ongoing research into optimizing β-glucan concentrations in food matrices, assessing long-term health effects, and exploring advanced processing techniques is crucial. In the cosmetic industry, further investigation into extraction methods, mechanisms of action for skin conditions, and synergistic effects with other skincare ingredients offers avenues for innovation.

The dynamic and multidisciplinary field of β-glucan research continues to offer a wealth of opportunities in nutrition, health, and industry. As we delve deeper into its molecular mechanisms, health benefits, and industrial applications, β-glucan is poised to play a pivotal role in enhancing human well-being and contributing to sustainable agriculture, food production, and healthcare practices. Ongoing and future studies in this area will continue to unveil the potential of β-glucan in shaping a healthier and more sustainable future.

## Figures and Tables

**Table 1:** Different sources of β-glucan and their molecular characterization.

Source	Common Name	M.wt (g/mol)	Linkage	Content	Solubility	Branching Frequency	DP3:DP4	References
*Triticum aestivum*	Wheat β-glucan	4.3–75.8 × 10^4^	β-1,3 and β-1,4	0.18–1.8 (%*w*/*w*)	Soluble		2.8–4.5	[9]
*Avena sativa*	Oat β-glucan	65–310 × 10^4^	β-1,3 and β-1,4	2.2–7.5 (%*w*/*w*)	Soluble		1.5–2.3	[10]
*Hordeum vulgare*	Barley β-glucan	31–270 × 10^4^	β-1,3 and β-1,4	2.2–19.8 (%*w*/*w*)	Soluble		1.8–3.4	[11]
*Sorghum bicolor*	Sorghum β-glucan	36 × 10^4^	β-1,3 and β-1,4	0.1–1.7 (%*w*/*w*)	Soluble		2.1–3	[12]
*Secale cereale*	Rye β-glucan	21–110 × 10^4^	β-1,3 and β-1,4	1.0–2.7 (%*w*/*w*)	Soluble		1.9–3	[11]
*Grifola frondosa*	Grifolan	3 × 10^4^	β-1,3 and β-1,6		Soluble	1/3	-	[13]
*Shizophyllan commune*	Schizophyllan	45 × 10^4^	β-1,3 and β-1,6		Soluble	1/3	-	[14]
*Lentinus edodus*	Lentinan	30–40 × 10^4^	β-1,3 and β-1,6		Soluble	2/5	-	[15]
*Saccharomyces cerevisiae*	Zymosan	24 × 10^4^	β-1,3 and β-1,6	5–7 (%*w*/*v*)	Insoluble	0.03–0.2	-	[16]
*Euglena gracilis*	Paramylon	50 × 10^4^	β-1,3	90 (%*w*/*v*)	Insoluble		-	[17]
*Alcaligenes faecalis*	Curdlan	10 × 10^4^	β-1,3			Unbranched	-	[18]

**Table 2:** Genes involved in the synthesis of β-Glucan in cereals.

Gene	Gene Source	Promoter	Host	Outcome	Refernces
*HvCslF4*	*Hordeum vulgare*	CaMV 35S	*Hordeum vulgare*	Increase in DP3/DP4 ratio and β-glucan in grain (upto 50%)	[24]
*HvCslF6*	*Hordeum vulgare*	CaMV 35S	*Nicotiana benthamiana*	Loss of the β-1,3 and β-1,4 linkage activity, hence lack of β-glucan in leaves	[25]
*HvCslF6* and *HvCslH1* *HvCslF3* and *HvCslF9*	*Hordeum vulgare*	OsU6snRNA	*Hordeum vulgare*	Lesser β-glucan, decrease in DP3:DP4 ratio, grain test weight decreased	[23]
*BdCSLF6*	*Brachypodium distachyon*	CaMV 35S	*Nicotiana benthamiana*	Alteration in carbon metabolism and reduction in grain β-glucan content	[26]
*OsCslF6*	*Oryza Sativa*	Cell wall specific promoter, CaMV 35S	*Arabidopsis*	Overexpression leads to accumulation of β-glucan and reduced growth	[27]
*OsCslF2*, *OsCslF4*	*Oryza Sativa*	CaMV 35S	*Arabidopsis*	Synthesize β-glucan in leaves	[28]
*TaCslF6*	*Triticum aestivum*	Endosperm specific	*Triticum aestivum*	30% reduction inβ-glucan in endosperm	[22]
*TaCslF6*	*Triticum aestivum*	CaMV 35S	*Nicotiana benthamiana*	Synthesize β-glucan in leaves	[29]

## Supplementary Materials

The following supporting information can be downloaded at: https://www.mdpi.com/article/10.3390/nu16060900/s1, Table S1: Genes involved in the synthesis of β-Glucan in yeast; Table S2: Different extraction methodologies developed and used to extract protein from the different sources of β-glucan. References [131,132,133,134,135,136,137,138,139,140,141,142] are cited in Supplementary File.
