# Supplementary material for: Beta-Glucan as a Soluble Dietary Fiber Source: Origins, Biosynthesis, Extraction, Purification, Structural Characteristics, Bioavailability, Biofunctional Attributes, Industrial Utilization, and Global Trade
Source: Nutrients. 2024 Mar 21;16(6):900. doi: 10.3390/nu16060900 (PMC10975496; doi:10.3390/nu16060900)
Supplement: Supplementary file 1 [file nutrients-16-00900-s001.zip › nutrients-2906774-supplementary.pdf]

Supplementary Table S1. Genes involved in the synthesis of β-Glucan in yeast.

Gene	Source	Encoded protein	References
cwg2+	*Schizosaccharomyces pombe*	β-subunit of a geranylgeranyltransferase type I	[131]
KRE6	*S. cerevisiae*	Type II membrane protein	[132]
PKC1	*S. cerevisiae*	Yeast homolog of the mammalian protein kinase C family	[133]
SKN1	*S. cerevisiae*	Membrane protein	[134]
LRG1	*S. cerevisiae*	RhoGAP protein	[135]

Supplementary Table S2. Different extraction methodologies developed and used to extract protein from the different sources of β-glucan.

Source	Extraction method	Procedure	Purification method	Reference
Barley	Aqueous extraction	100 g barley flour, 500 mL water	Centrifuge (10,000 rpm), 10 min, ethanol precipitate (95% ethanol)	[136]
Oats	Alkaline extraction	100 g oat bran, 100 mL 1% NaOH, 400 mL water	Centrifuge (10 min), ethanol precipitation (95% ethanol)	[137]
Oats	Enzymatic extraction	200 g oat bran, 20 mL β-glucanase enzyme solution (10 U/mL), 480 mL water	Centrifugation at 10,000 rpm for 10 minutes, ethanol precipitation (95% ethanol)	[138]
Barley	Ultrasound-assisted extraction	Barley flour, 500 mL water	Centrifugation at 13,000 rpm for 10 minutes, ethanol precipitation (95% ethanol)	[139]
Oats	Microwave-assisted extraction	100 g oat bran, 500 mL water	Centrifugation at 10,000 rpm for 10 minutes, ethanol precipitation (95% ethanol)	[140]
Mushrooms	Alkaline extraction	100 g mushrooms, 100 mL 1% sodium hydroxide solution, 400 mL water	Centrifugation at 10,000 rpm for 10 minutes, ethanol precipitation (95% ethanol)	[141]
Yeast	Enzymatic extraction	100 g yeast, 10 mL β-glucanase enzyme solution (10 U/mL), 490 mL water	Centrifugation at 10,000 rpm for 10 minutes, ethanol precipitation (95% ethanol)	[142]

References

1. Diaz, M.; Sanchez, Y.; Bennett, T.; Sun, C.R.; Godoy, C.; Tamanoi, F.; Duran, A.; Perez, P. The Schizosaccharomyces pombe cwg2+ gene codes for the beta subunit of a geranylgeranyltransferase type I required for beta-glucan synthesis. *EMBO J.* **1993**, *12*, 5245–5254. https://doi.org/10.1002/j.1460-2075.1993.tb06220.x.
2. Roemer, T.; Bussey, H. Yeast beta-glucan synthesis: KRE6 encodes a predicted type II membrane protein required for glucan synthesis in vivo and for glucan synthase activity in vitro. *Proc. Natl. Acad. Sci. USA* **1991**, *88*, 11295–11299. https://doi.org/10.1073/pnas.88.24.11295.
3. Levin, D.E.; Bartlett-Heubusch, E. Mutants in the S. cerevisiae PKC1 gene display a cell cycle-specific osmotic stability defect. *J. Cell Biol.* **1992**, *116*, 1221–1229. https://doi.org/10.1083/jcb.116.5.1221.
4. Roemer, T.; GParavicini; Payton, M.A.; Bussey, H. Characterization of the yeast (1→6)-beta-glucan biosynthetic components, Kre6p and Skn1p, and genetic interactions between the PKC1 pathway and extracellular matrix assembly. *J. Cell Biol.* **1994**, *127*, 567–579. https://doi.org/10.1083/jcb.127.2.567.
5. Watanabe, D.; Abe, M.; Ohya, Y. Yeast Lrg1p acts as a specialized RhoGAP regulating 1, 3-β-glucan synthesis. *Yeast* **2001**, *18*, 943–951. https://doi.org/10.1002/yea.742.
6. Maheshwari, G.; Sowrirajan, S.; Joseph, B. Extraction and isolation of β-glucan from grain sources—A review. *J. Food Sci.* **2017**, *82*, 1535–1545. https://doi.org/10.1111/1750-3841.13765.
7. Wood, P.J. Relationships between solution properties of cereal β-glucans and physiological effects—a review. Trends Food Sci. *Technol.* **2004**, *15*, 313–320. https://doi.org/10.1016/j.tifs.2003.03.001.
8. McCleary, B.V.; Codd, R. Measurement of (1→3),(1→4)-β-D-glucan in barley and oats: A streamlined enzymic procedure. *J. Sci. Food Agric.* **1991**, *55*, 303–312. https://doi.org/10.1002/jsfa.2740550215.
9. Sourki, A.H.; Koocheki, A.; Elahi, M. Ultrasound-assisted extraction of β-d-glucan from hull-less barley: Assessment of physicochemical and functional properties. *Int. J. Biol. Macromol.* **2017**, *95*, 462–475. https://doi.org/10.1016/j.ijbiomac.2016.10.111.
10. Harasym, J.; Olędzki, R. Comparison of conventional and microwave assisted heating on carbohydrate content, antioxidant capacity and postprandial glycemic response in oat meals. *Nutrients* **2018**, *10*, 207. https://doi.org/10.3390/nu10020207.
11. Chen, J.; Chen, L.; Lin, S.; Liu, C.; Cheung, P.C.K. Preparation and structural characterization of a partially depolymerized beta-glucan obtained from Poria cocos sclerotium by ultrasonic treatment. *Food Hydrocoll.* **2015**, *46*, 1–9. https://doi.org/10.1016/j.foodhyd.2014.12.005.
12. Yang, W.; Huang, G. Extraction methods and activities of natural glucans. *Trends Food Sci. Technol.* **2021**, *112*, 50–57. https://doi.org/10.1016/j.tifs.2021.03.025.
